# Atypical Signaling and Functional Desensitization Response of MAS Receptor to Peptide Ligands

**DOI:** 10.1371/journal.pone.0103520

**Published:** 2014-07-28

**Authors:** Kalyan C. Tirupula, Russell Desnoyer, Robert C. Speth, Sadashiva S. Karnik

**Affiliations:** 1 Department of Molecular Cardiology, Lerner Research Institute, Cleveland Clinic, Cleveland, Ohio, United States of America; 2 Department of Pharmaceutical Sciences, College of Pharmacy, Nova Southeastern University, Fort Lauderdale, Florida, United States of America; University of North Dakota, United States of America

## Abstract

MAS is a G protein-coupled receptor (GPCR) implicated in multiple physiological processes. Several physiological peptide ligands such as angiotensin-(1–7), angiotensin fragments and neuropeptide FF (NPFF) are reported to act on MAS. Studies of conventional G protein signaling and receptor desensitization upon stimulation of MAS with the peptide ligands are limited so far. Therefore, we systematically analyzed G protein signals activated by the peptide ligands. MAS-selective non-peptide ligands that were previously shown to activate G proteins were used as controls for comparison on a common cell based assay platform. Activation of MAS by the non-peptide agonist (1) increased intracellular calcium and D-*myo*-inositol-1-phosphate (IP1) levels which are indicative of the activation of classical Gα_q_-phospholipase C signaling pathways, (2) decreased Gα_i_ mediated cAMP levels and (3) stimulated Gα_12_-dependent expression of luciferase reporter. In all these assays, MAS exhibited strong constitutive activity that was inhibited by the non-peptide inverse agonist. Further, in the calcium response assay, MAS was resistant to stimulation by a second dose of the non-peptide agonist after the first activation has waned suggesting functional desensitization. In contrast, activation of MAS by the peptide ligand NPFF initiated a rapid rise in intracellular calcium with very weak IP1 accumulation which is unlike classical Gα_q_-phospholipase C signaling pathway. NPFF only weakly stimulated MAS-mediated activation of Gα_12_ and Gα_i_ signaling pathways. Furthermore, unlike non-peptide agonist-activated MAS, NPFF-activated MAS could be readily re-stimulated the second time by the agonists. Functional assays with key ligand binding MAS mutants suggest that NPFF and non-peptide ligands bind to overlapping regions. Angiotensin-(1–7) and other angiotensin fragments weakly potentiated an NPFF-like calcium response at non-physiological concentrations (≥100 µM). Overall, our data suggest that peptide ligands induce atypical signaling and functional desensitization of MAS.

## Introduction

MAS is a G protein-coupled receptor (GPCR) encoded by the proto-oncogene *MAS* [1]. Gene knockout studies have been instrumental in defining MAS function [2]. MAS knockout mice are reported to have an overall impairment in cardiac function and vascular homeostasis as a result of pro-fibrotic changes and endothelial dysfunction, respectively [3]–[8]. MAS deficient mice also exhibit renal and metabolic disorders, alterations in hemostasis and pathological changes in several other tissues and organs [9]–[14]. In contrast, MAS deficiency is shown to offer protection from salt induced hypertension and inhibiting MAS function is shown to prevent ischemia/reperfusion injury in both kidney and heart [15]–[18]. Therefore, MAS plays a key role in several physiological processes and is a potential target for development of novel therapeutics for multiple disorders.

MAS is the prototype for Mas-related GPCR subfamily consisting of mostly orphan GPCRs that bind neuropeptides and have key physiological functions [1], [19], [20]. MAS plays an important role in the renin-angiotensin system and its effects are believed to be mediated by its putative endogenous peptide ligand, angiotensin (1–7) (Ang(1–7)) [21]. Ang(1–7) was shown to stimulate arachidonic acid production in CHO and COS cells transfected with MAS and in human mesangial cells that express MAS [21]–[23]. Angiotensin peptide metabolites, AngIII and AngIV, were also reported to activate MAS in similar arachidonic acid release assays in COS cells [22]. Despite MAS being a GPCR, there are reports indicating the lack of activation of conventional G protein signaling pathways upon stimulation with Ang(1–7). For example, in MAS expressing cells the intracellular levels of the classical G protein induced second messenger molecules such as calcium, inositol 1,4,5-trisphosphate and cAMP were not altered upon Ang(1–7) treatment [17], [24], [25]. However, neuropeptide FF (NPFF), which is unrelated to angiotensin, was reported to activate G protein mediated calcium signaling in HEK293 cells transfected with MAS [19]. Several other synthetic peptide and non-peptide ligands have also been reported to activate MAS in different *in vitro* and *in vivo* assays (Table S1). Among these, the non-peptide ligands AR234960 (AR-*agonist*) and AR244555 (AR-*inverse agonist*) are relatively better characterized and are demonstrated to specifically act through MAS and modulate at least two different classical G protein mediated signaling pathways [17]. Based on these findings we hypothesize that functional selectivity and pleiotropic signaling are at play upon activation of MAS by different ligands. Therefore, in this study we systematically analyzed MAS-dependent activation of major G protein signaling pathways and subsequent functional desensitization of the receptor in response to physiological peptide ligands while using the MAS-selective non-peptide ligands (AR-*agonist* and AR-*inverse agonist*) as controls. This study is of significance as comprehensive pharmacological characterization of MAS signaling is essential for developing clinical therapeutics targeting MAS function.

To facilitate these studies, we established a tetracycline-inducible *myc*-tagged human MAS expression system in HEK293 cells. In this stable cell line, we optimized a panel of well-established commercially available fluorescence, FRET and luciferase based assays to measure activation of different G protein signaling pathways. We also evaluated re-stimulation of MAS following activation by different ligands in a modified fluorescence based calcium assay to assess functional desensitization of the receptor. We observed activation of multiple G protein signaling pathways by MAS both constitutively and in the presence of non-peptide agonist. The putative endogenous ligand, Ang(1–7) failed to activate any of the major G protein signaling pathways of MAS within the pharmacological concentration range while a unique and complex signaling profile was observed upon activation of MAS by NPFF. For the first time we demonstrate that activation of MAS by peptide ligands activates a panel of signaling pathways that are very different and less understood compared to the control non-peptide ligands. Our data confirms functional selectivity of peptide ligands towards both activation and functional desensitization of MAS. We propose a schematic model for ligand-receptor-effector coupling for MAS.

## Materials and Methods

### Secondary structure model for MAS and residue numbering scheme

**Figure 1:**
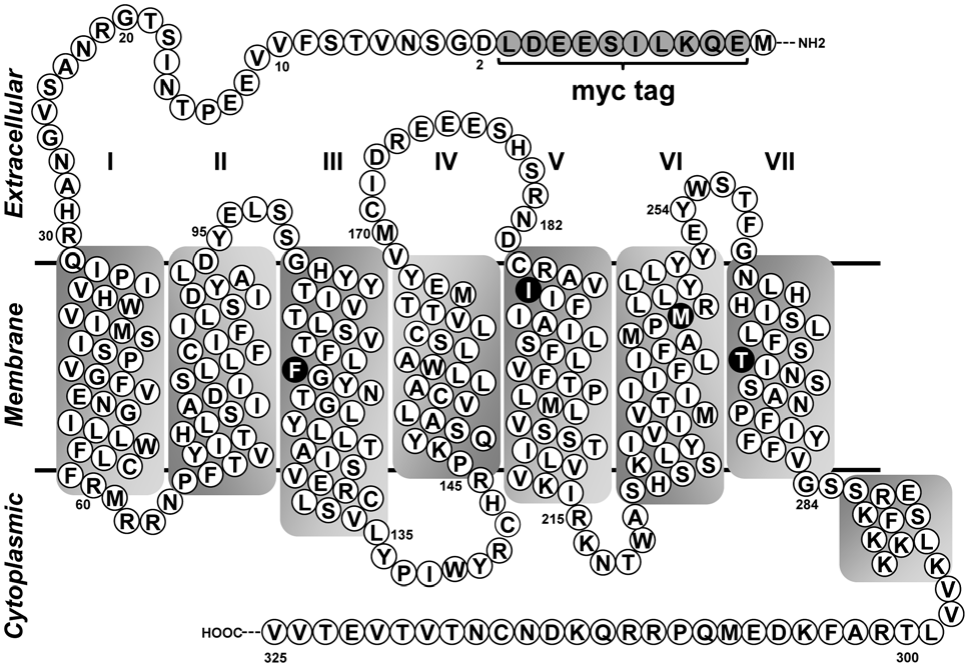
Secondary structure model of MAS highlighting the mutation sites. The *myc*-tag on the receptor and the locations of mutations used in the study are shown on the secondary structure model of MAS receptor. The transmembrane helices (TM) I - VII are predicted by bioinformatics analysis. Also shown in the model are the predicted ligand binding residues that were mutated in this study.

The secondary structure model for MAS (Figure 1) was generated by predicting the transmembrane (TM) boundaries for helices TM1-7 and for the non-TM ‘helix 8’ based on pair-wise sequence alignments with eight different GPCRs [rhodopsin (bovine and squid), β_1_-adrenergic, β_2_-adrenergic, adenosine A_2A_, C-X-C chemokine type 4, dopamine D_3_ and histamine H_1_ receptors] with known crystal structures at the time of this study.

The position of amino acids in human MAS shown in Figure 1 is based on its sequence as provided in the genbank record NP_002368 [26]. To facilitate comparison with residues at homologous positions in the TM regions of other GPCRs, the generic numbering proposed by Ballesteros and Weinstein is also mentioned as a superscript where required [27]. For example, residue F112 on TM3 in MAS, is also referred to as F112^3.32^ in the Ballesteros and Weinstein numbering. The homologous residues at this position in rhodopsin and Angiotensin II type 1 receptor (AT1R) are A117^3.32^ and V108^3.32^, respectively.

### Cloning of wild-type (WT) and mutant MAS receptors

The WT MAS expression construct was synthesized with an N-terminal *myc*-tag (Figure 1) by GenScript (Piscataway, NJ). The *myc*-tagged WT MAS from this vector was initially sub-cloned into pcDNA3.1 and later sub-cloned into tetracycline/doxycycline inducible vector pcDNA 5/TO (Life Technologies, Grand Island, NY). Four different ligand binding MAS mutants (F112A, I191A, M244A and T270A) were created using site-directed mutagenesis of WT *myc*-tagged MAS in pcDNA3.1 or pcDNA 5/TO vector background (Agilent Technologies, Santa Clara, CA). WT MAS receptor without an epitope tag was constructed by replacing the 5′ portion of the *myc*-tagged WT MAS in pcDNA 5/TO by untagged MAS sequence from a plasmid construct provided as a gift from Arena Pharmaceuticals, Inc. (San Diego, CA). The sequences of all WT and mutant MAS genes in plasmid constructs were verified by capillary DNA sequencing at the Genomics Core at Lerner Research Institute (Cleveland, OH).

### Pharmacological compounds

Non-peptide ligands AR234960 (AR-*agonist*) and AR244555 (AR-*inverse agonist*) were a gift from Arena Pharmaceuticals, Inc. (San Diego, CA). Neuropeptide FF (NPFF) was initially purchased from Bachem (King of Prussia, PA). NPFF and NPFF analogs [Y^1^]-NPFF, NPFF-C were synthesized by the Molecular Biotechnology Core at Lerner Research Institute (Cleveland, OH). The NPFF analog [^D^Y^1^] [^NMe^F^3^]-NPFF was obtained from Bachem (King of Prussia, PA). Ang(1–7) and angiotensin metabolites were obtained from multiple sources. Ang(1–7) was purchased from Bachem (King of Prussia, PA), Sigma-Aldrich (St. Louis, MO) and Phoenix Pharmaceuticals (Burlingame, CA). Ang(1–7)-amide was purchased from Labpe Chemicals (Houston, TX). Ang(1–7) analogs ([^D^A^7^]-Ang(1–7) (A779) and [Sar^1^]-Ang(1–7)-amide) and angiotensin metabolites (AngIII, AngIV/Ang(3–8) and Ang(3–7)) were purchased from Bachem (King of Prussia, PA). AngIV-amide was synthesized by the Molecular Biotechnology core at Lerner Research Institute (Cleveland, OH). Pan-inhibitor of G-protein signaling, BIM-46187, was a gift from IPSEN Innovation (Les Ulis, France).

### Preparation of stock solutions

Non-peptide ligands (AR-*agonist* and AR-*inverse agonist*) and peptide ligands were dissolved in DMSO and water, respectively, to prepare 10 mM stocks. BIM-46187 was dissolved in DMSO as a 10 mM stock. The pH of the buffer in the experiments was verified to be neutral (7–7.5) after adding the ligands at desired concentrations.

### Cell culture media and buffers

HEK293 cells were grown in complete media (DMEM supplemented with fetal bovine serum (10%), penicillin/streptomycin (100 units/ml). Wild-type and mutant MAS expressing stable cell lines were grown in complete media supplemented with blasticidin (5 µg/ml) and hygromycin (300 µg/ml). Cells were maintained in a humidified incubator at 37°C and 5% CO_2_. Stable cells were induced with complete media containing doxycycline (100 ng/ml) for 26–28 h. Un-induced controls in the experiments were maintained under similar conditions as induced cells but without addition of doxycycline.

### Establishment of stable cell lines

Tetracycline/doxycycline inducible stable cell lines for WT and mutant MAS were established in T-Rex HEK293 cells (Life Technologies, Grand Island, NY). Cells were maintained under blasticidin selection prior to transfection with WT or mutant MAS in pcDNA 5/TO. Stably transfected cells were selected using hygromycin (300 µg/ml) and individual clones were isolated and expanded. These cell lines were then induced and screened for maximal receptor expression. The desired clones were preserved as cryostocks and were de-banked as needed. For the WT stable cell line, the concentration of doxycycline and time for induction were optimized by screening different conditions. The optimized induction conditions of treating the cells with 100 ng/ml doxycycline for 26–28 h were used for experiments with both the WT and mutant MAS stable cell lines. Un-induced cells were used as negative controls in different assays performed in this study. The cell lines were continuously maintained in selection media and all experiments were performed with cells within 20 passages.

### Immunofluorescence and confocal microscopy

Immunostaining of cells was carried out 26–28 h post-induction. Cells plated on poly-L-lysine coated cover slips were washed with HBSS (Hank’s Balanced Salt Solution (HBSS): 0.44 mM KH_2_PO_4_, 0.34 mM Na_2_HPO_4_, 137 mM NaCl, 5.36 mM KCl, 1.26 mM CaCl_2_, 0.81 mM MgSO_4_, 0.5 mM MgCl_2_, 4.17 mM NaHCO_3_, 5.55 mM *D*-Glucose pH 7.3) and fixed with 4% paraformaldehyde. The cell membranes were stained by incubating cells with 5 µg/ml Alexa Fluor 555 conjugated wheat germ agglutinin (Life Technologies, Grand Island, NY) for 10 min at room temperature. Cells were permeabilized with 0.2% Triton X for 5 min to detect the total expression and spatial distribution of WT and mutant receptors in the cells. The cells were then blocked for 1 h in 3% bovine serum albumin (BSA). To detect MAS, cells were incubated overnight at 4°C with 1 µg/ml anti-c-*myc* (9E10) antibody conjugated to Alexa Fluor 488 (Santa Cruz Biotechnology, Inc. Santa Cruz, CA). Finally, the coverslips with immunostained cells were mounted on a glass slide with Vectashield mounting medium with DAPI (Vector Laboratories, Burlingame, CA). Confocal images were taken on a Leica TCS SP2 confocal fluorescence microscope (Imaging Core, Lerner Research Institute, Cleveland).

### Fluorescent Imaging Plate Reader (FLIPR) assay to measure calcium

The assay was performed using FLIPR Calcium 5 assay kit (Molecular Devices, Sunnyvale, CA). For calcium measurements, cells at a density of 125,000 cells/well in 100 µl medium were seeded onto a 96-well clear bottom black cell culture plate that was pre-coated with poly-L-lysine. In the calcium assays the cell density of 125,000 cells/well was consistently maintained to minimize variability between independent experiments. The cells were seeded in induction media to induce MAS expression or in complete media for negative controls. The plate was maintained in a cell culture incubator for 26–28 h. The cells were then serum starved for 2 h by replacing the medium with 100 µl of serum free DMEM. In the case of pre-treatment with pan-inhibitor of G protein signaling, BIM-46187, the cells were initially serum starved for 1 h by replacing the medium with 50 µl of serum free DMEM. After 1 h, 50 µl of BIM-46187 was added to the cells to a final concentration of 25 µM and treated for another 1 h. Following serum starvation, 100 µl of calcium sensitive dye along with 2x (2.5 mM final concentration) probenecid (Life Technologies, Grand Island, NY) was added to the cells. During this step, AR-*inverse agonist* or BIM-46187 was also added at desired concentrations to the calcium dye preparation in case of experiments where cells were pre-treated with these inhibitors. The cells were maintained for one hour in the cell culture incubator. Following this, the 96-well plate containing cells loaded with calcium dye and a U-bottom 96-well plate containing ligands at 5x the desired final concentration in D-PBS (1.47 mM KH_2_PO_4_, 138 mM NaCl, 2.67 mM KCl, 8.1 mM Na_2_HPO_4_, pH 7.3) were allowed to equilibrate for 15 min on a FlexStation 3 instrument (Molecular Devices, Sunnyvale, CA) at 37°C. The plates were further equilibrated on the instrument for 5 min with the lids removed from the plates. The instrument was programmed in FLEX mode to add ligands (50 µl at 5x concentration) to the cells and to monitor the fluorescence before and after adding the ligands. In re-stimulation and antagonism assays the instrument is programmed to add 35 µl of first ligand followed by addition of the second ligand (35 µl) after 10 min. It is important to note that there were solubility issues with the AR-*agonist* at greater than 50 µM concentration. Therefore, when using 5x ligand stock the maximum AR-*agonist* concentration that could be tested was 10 µM.

### Homogenous time-resolved fluorescence inositol-1-phosphate (IP1) assay

IP1 levels in the cells were measured using the IP-One Tb kit (Cisbio US, Bedford, MA). For the assay, cells were seeded onto a 384-well low volume white cell culture plate at a density of 25,000 cells/well in 20 µl of induction or complete medium. In the IP1 assays the cell density of 25,000 cells/well was consistently maintained to minimize variability between independent experiments. The cells were maintained in a cell culture incubator for 26–28 h. The cells were then serum starved for 2 h by replacing the medium with 7 µl of serum free DMEM/F-12. During this step, 7 µl of serum free DMEM/F-12 was also added to the wells to which IP1 standards would be subsequently added. Following serum starvation, 7 µl of ligands and serially diluted IP1-standards prepared at 2x concentration in stimulation buffer (10 mM Hepes, 1 mM CaCl_2_, 0.5 mM MgCl_2_, 4.2 mM KCl, 146 mM NaCl, 5.5 mM glucose, 50 mM LiCl, pH 7.4) were added to the appropriate wells. The cells were then placed in the cell culture incubator for 4 h. Later, 3 µl each of d2-labeled IP1 and anti-IP1 cryptate Tb conjugated antibody diluted in lysis buffer was added sequentially to all the wells and the plate was incubated overnight in the dark at room temperature. Time-resolved ratiometric fluorescence emission measurements at 665 nm and 620 nm were taken after excitation at 343 nm on FlexStation 3 instrument. In contrast to the calcium assays, the IP1 experimental conditions allowed for testing up to 50 µM of AR-*agonist* since the ligand stocks were made at 2x concentrations.

### Luciferase reporter assay

The luciferase assay was performed using pGL4 luciferase reporter vectors and Dual-Glo Luciferase Assay System (Promega, Madison, WI). For this assay, the inducible MAS stable cells were initially plated in a 10 cm plate and left overnight in a cell culture incubator. The following day the cells were transfected with 5 µg of pGL4.34 plasmid containing serum response factor response element (SRF-RE)-firefly luciferase reporter gene and 1 µg of pGL4.73 plasmid containing *renilla* luciferase (as a transfection efficiency control). The cells were also induced during transfection by adding doxycycline except for un-induced negative controls. After 8 h of transfection and induction, the cells were seeded onto a 96-well clear bottom black cell culture plate (pre-coated with poly-L-lysine) at a density of 125,000 cells/well in 100 µl of minimal medium (with reduced serum of 0.5%). The plate was maintained in a cell culture incubator for 18–20 h. At this point 50 µl of media in the cells was replaced with 50 µl of serum free media containing ligands at 2x the desired concentration and incubated for an additional 6 h. Finally, the luciferase activity in the cells was measured according the protocol provided by the manufacturer. The luminescence measurements (RLU) in the cells were obtained on FlexStation 3 instrument.

### Data analysis and statistics

In the calcium assays, the maximum response and minimum response values in relative fluorescence units (RFU) were extracted by subtracting the corresponding baseline value wherein baseline is defined as the average fluorescence of all time points before addition of ligand.

The kinetic parameter t_1/2_ (time taken to reach half of the maximum response) was determined using SoftMax Pro software supplied with the FlexStation 3 instrument (Molecular Devices, Sunnyvale, CA).

For re-stimulation assays, the data is expressed as the percentage of maximum agonist stimulation to allow for ready comparison between individual experiments. For the IP1 assay, a standard curve was generated using the 4-Parameter logistic equation fit of the HTRF readings of IP1 standards using the SoftMax Pro software. The IP1 concentrations in the experiments were extrapolated based on the standard curve parameters. In the luciferase assay, the luminescence measurements (RLU) are presented as a ratio of firefly to *renilla* luciferase activity. For all the assays, the EC_50_ and IC_50_ values were estimated by fitting the data to a three parameter dose response using GraphPad Prism software (GraphPad, La Jolla, CA).

All experiments were repeated at least three times (N≥3) under identical conditions and each experiment is performed in triplicate. In the figures, representative data from one independent experiment performed in triplicate is shown. Data in the tables is presented as mean±SEM wherein SEM is calculated from at least three independent experiments. A statistical analysis of the data was performed by unpaired Student’s *t*-test using GraphPad Prism software. Significance levels of *t*-test are given as: *p<0.05; **p<0.005; ***p<0.0001.

## Results

### Generation of mutant MAS receptors for this study

The choice of ligand binding residues to mutate was based on extensive review of structure-function relationship literature on rhodopsin (prototypical GPCR) and the Angiotensin II type 1 receptor (AT1R). The residues in transmembrane regions at positions 3.32, 5.42, 6.51 and 7.43 (Ballesteros-Weinstein numbering) in rhodopsin (A117^3.32^, M207^5.42^, Y268^6.51^ and K296^7.43^) and AT1R (V108^3.32^, K199^5.42^, H256^6.51^ and Y292^7.43^) are reported to be critical for binding native ligands in each receptor [28]–[40]. These residues are also part of the ligand binding pocket as observed in other GPCR structures [41], [42]. The corresponding residues in MAS were independently mutated to alanines (A) to result in F112A, I191A, M244A and T270A ligand binding mutants (Figure 1). These ligand binding mutants were used to test the hypothesis that the peptide and non-peptide ligands of MAS interact with these residues to exert their effects.

### Induced expression of wild-type (WT) and mutant MAS receptors

**Figure 2:**
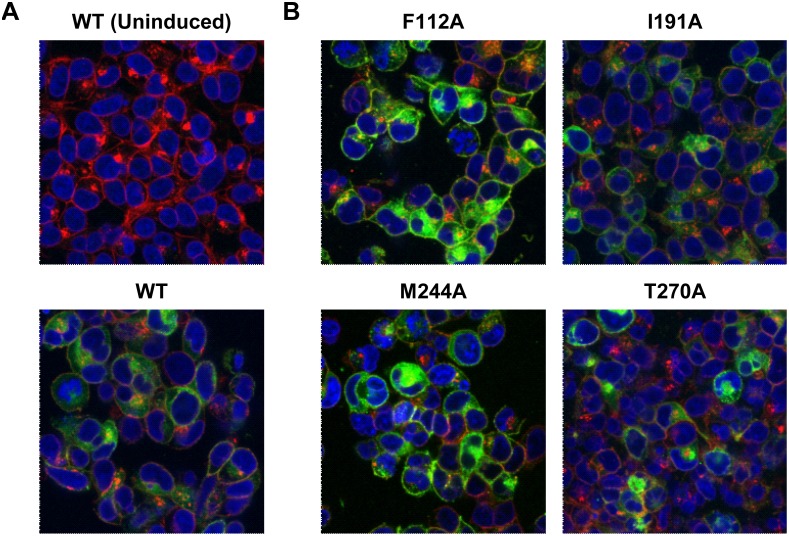
Expression of WT and mutant MAS in stable cell lines. Total expression of *myc*-tagged MAS was evaluated by (A and B) confocal microscopy. Images are labeled in green, red and blue for MAS, membranes and nuclei, respectively. (A) MAS expression is seen only in induced cells (bottom panel) compared to un-induced cells (top panel). (B) Expression of mutant MAS in ligand binding mutants. The anti-c-*myc* (9E10) antibody (Santa Cruz Biotechnology, Inc. Santa Cruz, CA) was used for imaging the receptor.

All MAS constructs used in this study have an N-terminal *myc-*tag (Figure 1). Total and cell surface expression of WT and mutant MAS was evaluated by confocal microscopy and whole cell ELISA, respectively. In the un-induced cells there was no detectable expression of WT MAS. Upon induction with doxycycline, WT MAS was strongly expressed and was localized both on the plasma membrane and intracellular compartments (Figure 2A). This distribution of WT MAS in the cells was similar to the findings previously reported for MAS-GFP constructs [25], [43]. Similar to the WT MAS, cell surface and intracellular localization was observed for all MAS mutant receptors (Figure 2B). Cell surface expression levels of mutants relative to the WT were quantified by whole-cell ELISA (Figure S1). The receptor quantitation from ELISA was used to compare the constitutive activity of the WT and mutant receptors.

### Constitutive activity in MAS and its modulation by AR-*agonist* and AR-*inverse agonist*

**Figure 3:**
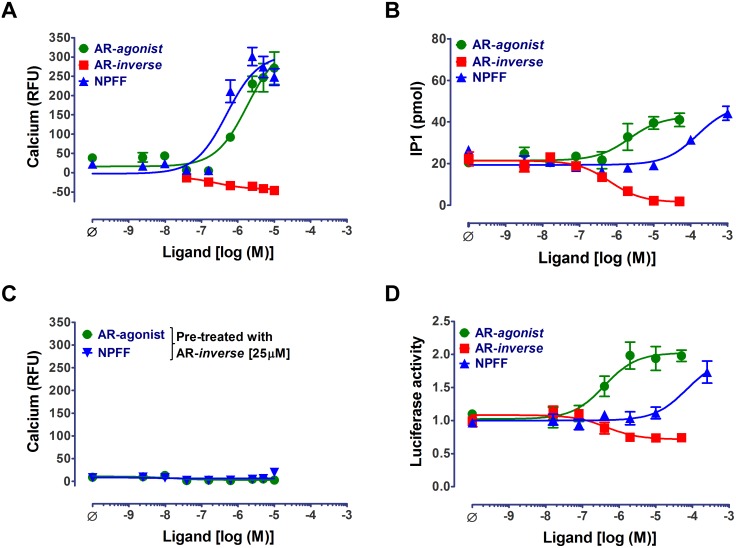
Calcium, IP1 and luciferase assay responses in WT MAS stable cell line. Dose dependent changes in (A) calcium flux and (B) IP1 levels in cells upon stimulation with AR-*agonist*, AR-*inverse agonist* (AR-*inverse*) and NPFF in induced WT stable cell line. (C) Complete inhibition of AR-*agonist* and NPFF dose-response curves upon pre-treatment with 25 µM of AR-*inverse agonist* (AR-*inverse*) in calcium assays. (D) Dose dependent changes in luciferase expression upon stimulation with AR-*agonist*, AR-*inverse agonist* (AR-*inverse*) and NPFF in induced WT stable cell line. Representative curves from a single experiment wherein measurements are made in triplicate are shown as mean±SEM. The number of independent experiments is: N> = 3 in panels A, B and D; N = 2 in panel C.

The basal calcium levels in induced cells were significantly higher (Figure S2A) suggesting basal/constitutive activity of the receptor. Similarly, the basal IP1 levels in the WT MAS induced cells were also significantly higher than those of un-induced controls (Figure S2B and Table S2). AR-*agonist* treatment further increased the intracellular calcium and IP1 levels in a dose-dependent manner with similar EC_50_ values in induced cells (Figure 3A and 3B; Table 1). The AR-*inverse agonist* inhibited the elevated calcium and IP1 levels in induced cells in a dose-dependent manner confirming the constitutive activity of WT MAS.

**Table 1:** Summary of IC_50_ and EC_50_ values for different ligands in multiple functional assays.

	EC_50_ or IC_50_ (µM)		
Ligand	Calcium assay	IP1 assay	Luciferase assay
**AR-** ***inverse***	0.9±0.6	0.7±0.1	0.4±0.1
**AR-** ***agonist***	1.5±0.3	1.3±0.3	0.5±0.1
**NPFF**	0.4±0.1	>100	>50
**Ang(1–7)**	*cbd*	*cbd*	*cbd*

Values are mean±SEM from at least three independent experiments; *cbd* = cannot be determined.

Pre-treatment of cells with AR-*inverse agonist* caused a rightward-shift of the dose-dependent calcium response curves for AR-*agonist* with complete inhibition observed at 25 µM suggesting competitive antagonism (Figure 3C). The modulation of both calcium and IP1 levels in the cells along with the inhibitory effect of phospholipase C (PLC) inhibitor (U73122) in calcium dose-response curves (data not shown) suggested the involvement of Gα_q_-PLC mediated signaling by MAS.

Additionally, in Gα_12_-dependent expression of luciferase reporter assay we observed significant constitutive activity of MAS as measured by the ratio of firefly to *renilla* luciferase activity (Figure S2C). The AR-*agonist* promoted luciferase expression beyond the constitutive activity while AR-*inverse agonist* antagonized luciferase expression in a dose-dependent manner (Figure 3D; Table 1). We also observed elevated cAMP levels in induced cells compared to un-induced controls suggesting constitutive activation of Gα_s_ by MAS (Figure S2D). AR-*agonist* treatment decreased cAMP levels in induced cells suggesting involvement of Gα_i_ mediated signaling (data not shown) in line with previous studies [17]. In all the assays, the effects of both AR-*agonist* and AR-*inverse agonist* were specific to MAS in the induced cells and had no effect on un-induced cells (Figure S3A–S3C).

Therefore, our data along with reports from previous studies suggest that MAS constitutively activates major G protein signaling pathways and this activation is further enhanced or inhibited by the addition of AR-*agonist* or AR-*inverse agonist*, respectively.

### NPFF activation profile of MAS is distinct from AR-*agonist*

The neuropeptide hormone NPFF potently increased calcium levels with an EC_50_ value of 0.4±0.1 µM (Figure 3A; Table 1); this result is consistent with a previous study [19]. Several previously described structural analogs of NPFF [44]–[46] that were tested in the calcium response assay showed that MAS receptor activation is sensitive to the modification of the NPFF-peptide sequence (Figure S4; Table 2). Interestingly, stimulating the cells with 10 µM of NPFF (20 fold higher than the EC_50_) resulted in the maximal increase in calcium levels with no detectable increase in IP1 levels. NPFF treatment resulted in elevated IP1 levels at higher concentrations with an apparent EC_50_ value>100 µM (Figure 3B; Table 1). This activation profile of NPFF is different from that of the AR-*agonist* which increased both calcium and IP1 levels in the cells with similar EC_50_ values. Treatment with the AR-*inverse agonist* caused a rightward-shift and complete inhibition of NPFF dose-response in calcium assay (Figure 3C). Discordant EC_50_ values for calcium and IP1 production suggests that the two responses evoked by NPFF acting on MAS are partly independent and not sequential as anticipated from conventional G_q_-PLC signaling. Similar to IP1 dose-response, NPFF treatment activated Gα_12_-dependent expression of luciferase reporter only poorly (Figure 3D; Table 1). These findings suggest that the efficiency of NPFF in G protein signaling assays is different compared to AR-*agonist*. In all the assays, NPFF induced responses were absent in un-induced cells demonstrating that these are MAS specific signals (Figure S3A–S3C).

**Table 2:** NPFF and its analogs along with corresponding EC_50_ values in calcium assays.

		Calcium assay	
Peptide	Sequence	EC_50_ (µM)	Fold change
**NPFF** †	FLFQPQRF-amide	0.4±0.1	1
**[Y^1^]-NPFF**	YLFQPQRF-amide	12.2±2.0	30.5**
**[^D^Y^1^] [^NMe^F^3^]-NPFF**	^D^YL^NMe^FQPQRF-amide	2.4±0.3	6.0**
**NPFF-C**	FLFQPQRF	3.0±0.3	7.5**

† Physiological peptide; Values are mean±SEM from at least three independent experiments; Statistical significance (t-test) - *p<0.05, **p<0.005, p<0.0001.

The role for the cognate receptors for NPFF, neuropeptide FF receptor 1 (NPFFR1) and receptor 2 (NPFFR2), in the observed NPFF signaling in our system is expected to be negligible as both these receptors are reported to be poorly expressed in HEK293 cells [47]. We experimentally evaluated the expression of NPFF receptors in our stable cell line by real-time quantitative PCR. In the parental HEK293 cell lines, *NPFFR1*, *NPFFR2* and *MAS* were not expressed at significant levels (C_t_ values >29). Furthermore, there was no significant change in the mRNA expression levels of *NPFFR1* and *NPFFR2* in our inducible cell system under both un-induced and induced conditions (Figure S5). In contrast, the transcript levels for *MAS* were significantly higher in the induced cells while the expression levels were slightly elevated in the un-induced cells suggesting leaky expression.

**Figure 4:**
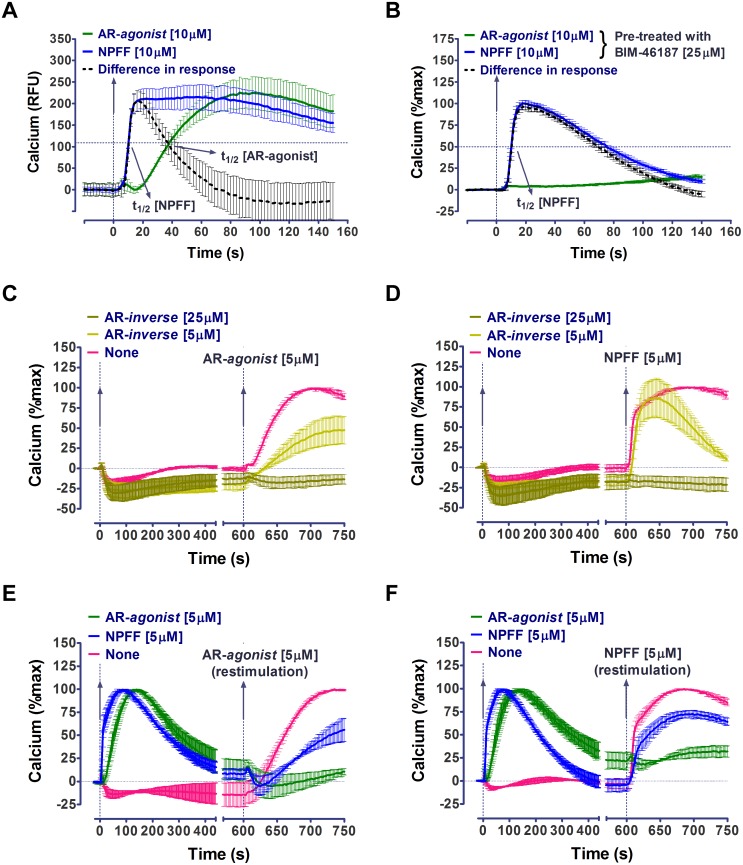
Differential MAS signaling upon treatment with AR-*agonist* and NPFF in calcium assays. The distinct signaling profiles of AR-*agonist* and NPFF are evident in (A and B) calcium flux kinetics, (C and D) antagonism and (E and F) re-stimulation assays. Calcium flux kinetics upon treatment with AR-*agonist* (in green) and NPFF (in blue) in the (A) absence and (B) presence of BIM-46187. The horizontal dashed line indicates the approximate fluorescence value which is half of the maximum observed upon ligand treatment. The kinetic parameter (t_1/2_) which is time taken to reach half of the maximal calcium response is also indicated for both ligands. This AR-*agonist* calcium response was subtracted from that of NPFF to highlight (in black and dashed line) the faster component in NPFF treated cells. In the antagonism assays the cells were initially treated with AR-*inverse agonist* (AR-*inverse*) at 25 µM (in dark olive green), 5 µM (in light olive green) and no ligand control (in magenta) and then challenged with (C) AR-*agonist* or (D) NPFF. In the re-stimulation assays the cells were initially treated with AR-*agonist* (in green), NPFF (in blue) and no ligand control (in magenta) and then challenged with (E) AR-*agonist* or (F) NPFF. In all the panels, vertical dashed lines indicate addition of ligands that are added at t = 0s and also at t = 600s in antagonism and re-stimulation assays. Data in B to F is normalized to maximum calcium response in the presence of agonists, AR-*agonist* or NPFF. Representative curves from a single experiment wherein measurements are made in triplicate (duplicate in case of antagonism and re-stimulation assays) are shown as mean±SEM. The number of independent experiments is N> = 3 in all the panels.

Further differences in MAS activation by NPFF and AR-*agonist* were evident in the kinetics of calcium flux. The calcium flux kinetics, measured as the time taken to reach half of the maximal calcium levels (t_1/2_), were significantly faster in the presence of NPFF (t_1/2_ = 8.5±0.3s) compared to AR-*agonist* (t_1/2_ = 31.2±2.1s) (Figure 4A). The calcium flux observed upon NPFF stimulation appears to be biphasic with an initial faster component and an AR-*agonist-*like slower component. This faster component in the calcium flux stimulated by NPFF was more apparent upon subtracting the AR-agonist calcium response from that of NPFF. When cells were pre-treated with 25 µM pan-inhibitor of G protein signaling, BIM-46187 [48], the fast component was selectively retained as seen in figure 4B. In AR-*agonist* treated cells there is complete loss of signal. Taken together these results suggest a G protein independent origin of the fast component of the NPFF-induced calcium signal.

In antagonism assays, wherein the first ligand was the AR-*inverse agonist*, the response of AR-*agonist* was antagonized in a dose-dependent manner as expected (Figure 4C). In a similar assay, 25 µM AR-*inverse agonist* completely inhibited NPFF response, however, initial treatment with 5 µM of AR-*inverse agonist* followed by re-stimulation with 5 µM NPFF resulted in a calcium flux that lacked the slower component but selectively retained the component with faster kinetics. This experiment suggests that the faster component of the calcium response to NPFF is not sensitive to AR-*inverse agonist* (Figure 4D), which may be another indication of G_q_-independence of this signal. These findings highlight further marked differences in the activation profiles of AR-*agonist* and NPFF.

Overall, NPFF (1) poorly activates major G protein signaling pathways through MAS and (2) strongly stimulates intracellular calcium release through the activation of a Gα_q_-PLC independent pathway.

### NPFF activated MAS but not AR-*agonist* activated MAS can be re-stimulated

To further characterize differences between the peptide and non-peptide, NPFF and AR-*agonist,* respectively, we designed re-stimulation assays in presence of different ligands. In figures 4E and 4F, the cells were first activated by an agonist at a higher concentration followed by the addition of a second agonist after the response to the first stimulation returned to baseline. In these assays, both the AR-*agonist* and NPFF produced signals when the cells were first treated with NPFF. However, the cells stimulated initially with AR-a*gonist* did not respond to a second stimulus with either NPFF or AR-*agonist*. Lack of re-stimulation with a second agonist after initial activation of MAS with AR-*agonist* probably suggests rapid functional desensitization of the receptor, a typical signaling behavior of classical GPCRs. The re-stimulation of NPFF-activated MAS with NPFF or AR-*agonist* with minimal loss in signal suggests lack of functional desensitization of MAS. These findings (1) confirm the poor efficacy of NPFF to stimulate MAS-mediated G protein signaling and (2) suggest the possibility of a G protein independent component in calcium signaling, both of which could lead to poor desensitization of the receptor.

### NPFF and non-peptide ligands show overlapping interaction with ligand binding residues of MAS

All the ligand binding mutants showed constitutive activity (Table S2). After normalizing the basal IP1 levels to cell surface receptor expression, the constitutive activity of F112A and M244A was significantly higher than WT (Figure S6). The constitutive activity of I191A mutant was slightly but significantly lower than that of WT, while the constitutive activity of T270A mutant was not significantly different from WT.

**Figure 5:**
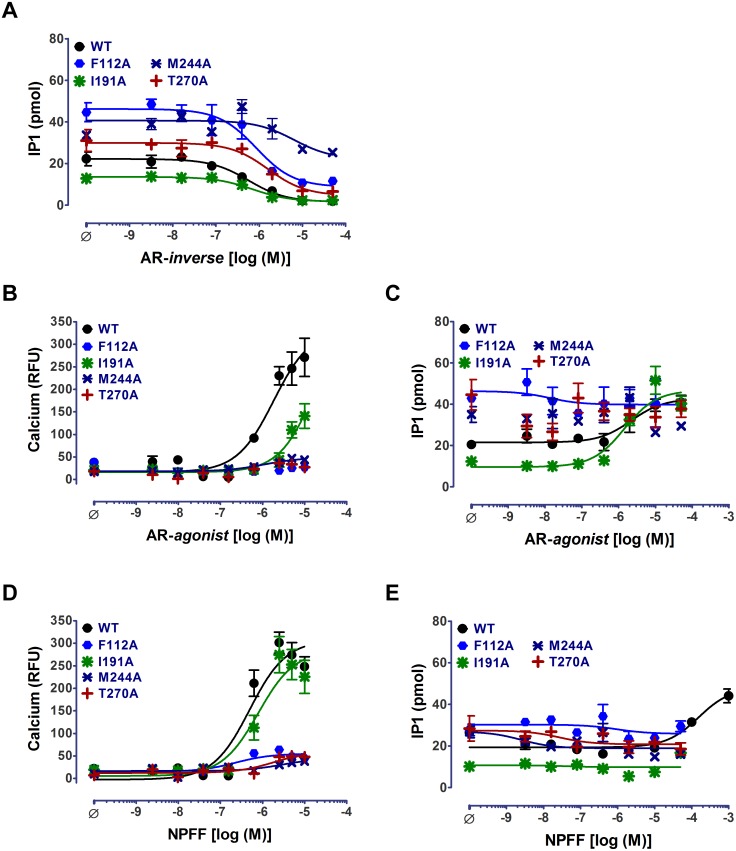
Calcium and IP1 signaling in ligand binding MAS mutants. Dose-response curves for (A) AR-*inverse agonist* (AR-*inverse*) treatment in ligand binding domain mutant MAS stable cell lines. Dose response curves for (B, C) AR-*agonist* and (D, E) NPFF treatment measured as function of intracellular (B, D) calcium and (C, E) IP1 levels. Representative curves from a single experiment wherein measurements are made in triplicate are shown as mean±SEM. The number of independent experiments is N> = 3 in all the panels.

The constitutive activity of all mutants except for the M244A was potently inhibited by AR-*inverse agonist* treatment (Figure 5A; Table 3). Incomplete inhibition of constitutive activity in the M244A mutant suggests that it is an important residue for the AR-*inverse agonist* interaction with MAS. M244A mutant also failed to respond to the treatment with AR-*agonist* and NPFF in both IP1 and calcium response assays, suggesting that both types of agonists require interaction with M244 to activate MAS (Figure 5B–5E; Table 3). The activation of calcium flux induced by NPFF in I191A mutant was similar to WT, while the AR-*agonist* induced response was significantly weaker (12-fold increase in EC_50_). These observations imply that interaction of I191 with AR-*agonist* is essential for activation but not critical for activation by NPFF. In the F112A and T270A mutants the activation response to AR-*agonist* and NPFF was defective; both mutants responded weakly but differentially in calcium assays (Figure 5B and 5D; See t_1/2_ values in Table 3). Only, the I191A mutant had WT-like activation profile in the IP1 assays (Figure 5C and 5E; Table 3). The IP1 levels in un-induced cells treated with ligands were unchanged except for M244A and T270A mutant stable cell lines. These mutants responded very weakly suggesting slightly leaky expression of the receptor in these mutants (Figure S7).

**Table 3:** EC_50_ and t_1/2_ values for different ligands in WT and mutant MAS stable cell lines.

	AR-*inverse*		AR-*agonist*					NPFF		
	IP1 assay		IP1 assay		Calcium assay			Calcium assay		
MAS Constructs	IC_50_ (µM)	Fold overWT	EC_50_ (µM)	Fold overWT	EC_50_ (µM)	Fold overWT	t_1/2_ (s)	EC_50_ (µM)	Fold overWT	t_1/2_ (s)
**WT**	0.7±0.1	1	1.3±0.3	1	1.5±0.3	1	31.2±2.1	0.4±0.1	1	8.5±0.3
**Ligand binding mutants**										
**F112A**	2.1±0.4	3**	*cbd*	–	*cbd*	–	*cbd*	*cbd*	–	7.7±2.9^#^∧
**I191A**	0.9±0.1	**1.3**	2.4±1.1	**1.8**	17.6±3.8	12**	68.3±5.3	0.8±0.03	2*	9.0±0.4
**M244A**	*cbd*	–	*cbd*	–	*cbd*	–	*cbd*	*cbd*	–	*cbd*
**T270A**	1.7±0.03	2.5**	*cbd*	–	*cbd*	–	40.0±1.2^#^	*cbd*	–	9.3±2.0^#^∧

Values are mean±SEM from at least three independent experiments; ∧Data from two independent experiments; ^#^Very weak response (<100 RFU); *cbd* = cannot be determined; Statistical significance (t-test) - *p<0.05, **p<0.005, ***p<0.0001.

Overall our observations suggest that both non-peptide (AR-*agonist* and AR-*inverse agonist*) and peptide (NPFF) ligands differentially interact with the four residues in the canonical GPCR ligand binding pocket in MAS (see discussion).

### Atypical activation of calcium response by Ang(1–7) treatment of MAS

**Figure 6:**
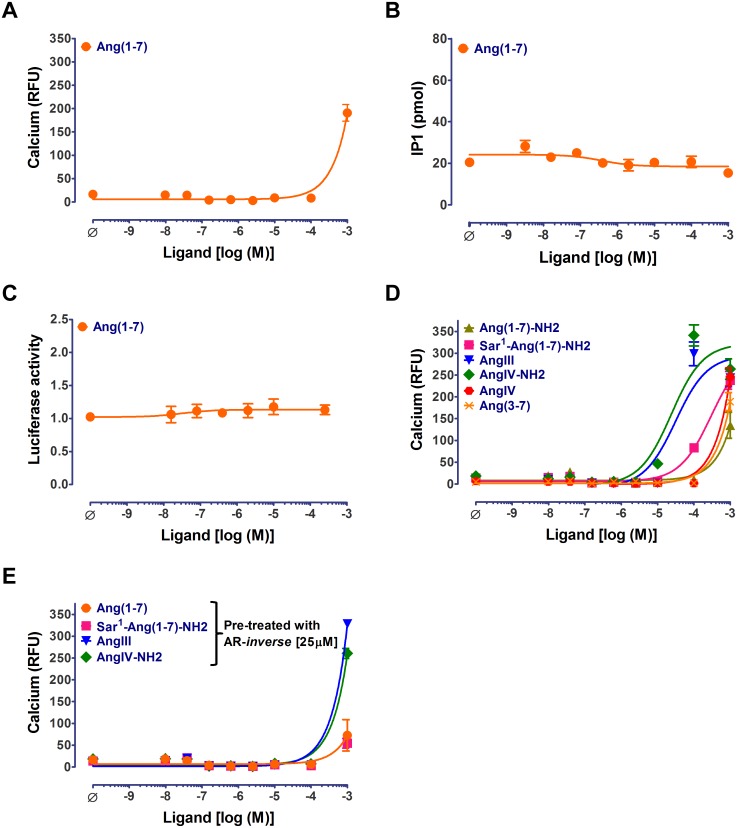
Evaluating response of Ang(1–7), Ang(1–7) analogs and angiotensin metabolites in MAS expressing stable cell lines. Ang(1–7) dose-response curves for MAS stable cell line in (A) calcium (B) IP1 and (C) luciferase assays. (D) Dose-response curves for Ang(1–7) analogs (Sar^1^-Ang(1–7)-NH2 and Ang(1–7)-NH2) and Angiotensin metabolites (AngIII, AngIV, AngIV-amide and Ang(3–7)) in calcium assays. (E) Inhibition of Ang(1–7), Sar^1^-Ang(1–7)-NH2 and AngIV-amide dose-response curves upon pre-treatment with 25 µM of AR-*inverse agonist* (AR-*inverse*) in calcium assays. Representative curves from a single experiment wherein measurements are made in triplicate are shown as mean±SEM. The number of independent experiments is: N> = 3 in panels A, B and C; N> = 2 in panel D; N> = 1 in panel E.

The generally believed MAS agonist, Ang(1–7), failed to activate MAS within the pharmacological concentration range in any of the functional assays (Figure 6A–6C; Table 1). We tested Ang(1–7) that was obtained from three different commercial sources to rule out any source specific artifacts. At 1 mM concentration, Ang(1–7) produced a significant calcium release with relatively faster kinetics (t_1/2_ = 11.8±0.6s; Table 4) that were comparable to that of NPFF. The activation of calcium response by Ang(1–7) observed at 1 mM concentration was inhibited by the AR-*inverse agonist* (Figure 6E) and was not observed in un-induced cells (Figure S3A) demonstrating that the response is specific to MAS. However, both IP1 and Gα_12_-driven luciferase signals were not detected at the highest concentrations of Ang(1–7) that were tested. These observations demonstrate that the low efficacy calcium response is atypical.

**Table 4:** EC_50_ and t_1/2_ values for Ang(1–7), Ang(1–7) analogs and angiotensin metabolites.

		Calcium assay	
Peptide	Sequence	EC_50_ (µM)	t_1/2_ (s)
**Ang(1–7)** †	DRVYIHP	*cbd*	11.8±0.6##
**A779**	DRVYIH^D^A	*cbd*	*cbd* #
**Ang(1–7)-amide**	DRVYIHP-amide	*cbd*	11.4±1.0##
**[Sar^1^]-Ang(1–7)-amide**	[Sar]RVYIHP-amide	193±59	18.2±1.2#
**AngIII** †	RVYIHPF	30.7±0.9	9.2±0.6#
**AngIV-amide**	VYIHPF-amide	24.4±1.5	13.7±0.9#
**AngIV / Ang(3–8)** †	VYIHPF	*cbd*	10.4±0.3##
**Ang(3–7)** †	VYIHP	*cbd*	15.0±0.5##

† Physiological peptide; Values are mean±SEM from at least three independent experiments.

# t_1/2_ at 100 µM ligand concentration;

## t_1/2_ at 1000 µM ligand concentration;

*cbd* = cannot be determined;

It is conceivable that the N-terminal *myc*-tag on our MAS construct interferes with the binding and signaling by Ang(1–7). Therefore, we cloned untagged WT MAS (see methods) and established an inducible HEK293 stable cell line. In this stable cell line we repeated calcium assays with Ang(1–7) along with NPFF and synthetic ligands as controls (Figure S8). Similar to the *myc*-tagged WT MAS stable cell line, Ang(1–7) up to 250 µM concentration showed no response in this new cell line while AR-*agonist*, NPFF, and AR-*inverse agonist* signaled as expected. Our data conclusively shows that the N-terminal *myc*-tag does not interfere with MAS signaling.

### MAS-mediated atypical calcium response to Ang(1–7) analogs and angiotensin fragments

We tested the ability of several Ang(1–7) structural analogs and angiotensin metabolites shown in table 4 to activate MAS. Previously, angiotensin fragments AngIII and AngIV were reported to activate MAS in an arachidonic acid release assay in COS cells [22].

To evaluate the efficacy of Ang(1–7) analogs and angiotensin metabolites in a dynamic assay, we modified the calcium assay re-stimulation protocol for simultaneous identification of both agonists and antagonists from a single screen as described previously [49]. MAS expressing cells were first treated with test ligands followed by re-stimulation with 5 µM NPFF. The re-stimulation of MAS was expected to be completely blocked in the event that the test ligand was an antagonist or inverse agonist. If the test ligand was an agonist, then MAS would be activated and the subsequent re-stimulation by NPFF would depend on the activation profile of the test ligand. For example, NPFF-like ligands would promote, while AR-*agonist*-like ligands would inhibit re-stimulation. If the test ligand does not modulate MAS function then re-stimulation with NPFF would result in normal activation of MAS.

Up to 10 µM concentration, peptides listed in table 4 did not produce any response and also did not interfere with re-stimulation by NPFF. At 100 µM concentration MAS was strongly activated by AngIV-NH2 and AngIII and weakly by Sar^1^-Ang(1–7)-NH2 (Figure S9). After initial activation with AngIV-NH2, AngIII and Sar^1^-Ang(1–7)-NH2, NPFF re-stimulated MAS, indicating lack of desensitization of MAS by these ligands.

Dose-response and AR-*inverse agonist* inhibition curves shown in figures 6D and 6E further characterized the efficacy of these peptides. At 1 mM concentration, most of the peptides stimulated calcium release with relatively faster kinetics that was comparable to that of NPFF (Table 4). The Sar^1^-Ang(1–7)-NH2 stimulated calcium release in a dose-dependent manner and this response was completely inhibited by AR-*inverse agonist* pre-treatment. The effect of Sar^1^-Ang(1–7)-NH2 was absent in un-induced cells suggesting MAS-specificity for this ligand (Figure S3D). In contrast, the calcium response produced by AngIII and AngIV-NH2 were incompletely inhibited by AR-*inverse agonist* pre-treatment. These peptides also produce non-specific calcium response in un-induced cells at 1 mM concentration. Thus, the portion of calcium response inhibited by AR-*inverse agonist* pre-treatment in MAS expressing cells very likely represents atypical activation of MAS by AngIII and AngIV-NH2. Overall our data suggests that angiotensin fragments and Ang(1–7) analogs are capable of activating MAS at very high (non-physiological) concentrations. The calcium response kinetics and re-stimulation profiles for these ligands are comparable to that of NPFF.

## Discussion

The importance of MAS was delineated through in vivo studies using MAS deficient mice in which physiological responses to Ang(1–7) peptide was lacking. Physiological peptides including NPFF and other angiotensin metabolites elicit second messenger responses (Table S1) from MAS but G protein dependence of MAS signaling was not examined in most studies. Recently, non-peptide ligands, AR-*agonist* and AR-*inverse agonist*, were shown to modulate MAS mediated Gα_q_ and Gα_i_ protein activation [17]. Whether peptide ligands of MAS activated classical G protein signaling pathways remained a question. In the present study, by comparing non-peptide ligand activated G protein signaling and functional desensitization of MAS to those by physiological peptide ligands, we discovered novel atypical pharmacological characteristics of human MAS receptor.

We established MAS expressing stable cell lines and optimized functional assays to study G protein activation by (1) measuring intracellular second messengers such as calcium, IP1, cAMP and (2) luciferase reporter gene expression under suitable Gα_12_ responsive element. We found that MAS is constitutively coupled to signaling pathways mediated by the G proteins, Gα_q_, Gα_i/s_ and Gα_12_. Previously, constitutive activity of MAS through only Gα_q_ was reported [17], [43]. The non-peptide ligands AR-*agonist* and AR-*inverse agonist* acted as anticipated. We confirmed AR-*agonist* promoted Gα_q_ and Gα_i_ activation as shown previously [17]. The MAS mediated (1) constitutive elevation of cAMP and (2) activation of Gα_12_ is reported here for the first time. AR-*inverse agonist* inhibited both the constitutive and agonist activated signaling pathways. The non-peptide ligand elicited MAS signaling was therefore analogous to classical GPCR signaling and functional desensitization.

The neuropeptide, NPFF, elicited MAS signaling profile was complex. It was different from the non-peptide agonist activation in the following ways: (1) weak activation of G protein signaling in multiple functional assays with the exception of calcium assay; (2) kinetics of calcium mobilization consisted of two components, an initial faster phase followed by a slower phase that resembled AR-*agonist* activated calcium response and (3) poor functional desensitization of the receptor. The characteristic two-component calcium signal produced by NPFF may be produced by dual mechanisms is emphasized by: (1) the release of calcium with an EC_50_ of 0.4 µM while the IP1 production with an EC_50_ of >1 mM suggesting a calcium release mechanism not requiring IP1 production in cells (Gα_q_-PLC independent?); (2) 10 µM of NPFF induces maximal calcium release without any increase in IP1 levels (Gα_q_-PLC independent?); (3) 25 µM of pan-inhibitor of G protein signaling selectively abolished the slower component and (4) pre-treatment with 5 µM non-peptide inverse agonist selectively inhibited the slower component (AR-*agonist* like, Gα_q_-PLC dependent?) and not the faster component of calcium response. For the first time, these studies demonstrate that NPFF poorly activates MAS-mediated G protein signaling but strongly potentiates atypical calcium signaling that possibly arises due to dual effector coupling by MAS. NPFF and other related RFamide peptides have been reported to be ligands for several other members of the MAS-related G-protein coupled receptor (MRG) subfamily based on calcium assays in heterologous cells [19], [20], [50]. MRGs such as mouse MRGA3 and human MRGX1 are reported to functionally interact with transient receptor potential (TRP) channels and modulate the calcium influx in sensory neurons [51], [52]. It is plausible that MAS activates TRP channels or other effector proteins capable of calcium release for its function suggesting a common mode of atypical activation similar to MRGs. The mechanistic basis for the absence of subsequent desensitization of peptide activated MAS receptor is not clear, but lack of G protein activation is expected to limit GRK-mediated desensitization. There is also currently no information available on the phosphorylation status of MAS receptor. The C-terminal tail sequence of MAS does not contain clusters of phosphorylatable Ser/Thr residues that are typical of other well-studied GPCRs. Interestingly, MRG subfamily members such as human MRGX1 and human MRGX2 were also shown to be resistant to phosphorylation, desensitization and endocytosis when activated by peptide ligands [53], [54].

The important question at this stage was whether the observed differences in NPFF and AR-*agonist* mediated signaling arise because of peptide and non-peptide ligands interacting with MAS at distinctly different sites. Structural analogs of NPFF (Table 2) elicited atypical calcium response and poor G protein activation. Modifying the peptide ligand sequence systematically altered efficacy of response and the change in EC_50_ for analogs validated the specificity of interaction with MAS. Establishing this structure-function principle was useful to evaluate other potential ligands of MAS, for instance Ang(1–7).

Functional analysis of predicted ligand binding pocket MAS mutants demonstrated that NPFF and the non-peptide ligands share an overlapping but not identical pocket. Constitutive activity of mutants F112^3.32^A, I191^5.42^A and T270^7.43^A was inhibited by AR-*inverse agonist* treatment while M244^6.51^A mutant responded only weakly. This mutant also lacked response to AR-*agonist* and NPFF treatment. M244^6.51^ appears to be differentially interacting with peptide and non-peptide agonists. M244^6.51^ may also distinguish between non-peptide agonist and inverse agonist binding. The homologous residue in AT1R (H256^6.51^) and rhodopsin (Y268^6.51^) are known to make stacking interactions with their respective ligands [28]–[30]. The reorientation of this residue is known to be important for receptor activation [31], [32]. The F112^3.32^ and T270^7.43^ are essential for agonist interaction since mutants F112^3.32^A and T270^7.43^A showed defects in agonist-activation. While both mutants show calcium response to NPFF treatment, only T270^7.43^A showed detectable calcium signal to AR-*agonist* treatment. The homologous residue for F112^3.32^ in rhodopsin (A117^3.32^) and AT1R (V108^3.32^) is known to be important for the stability of activated state of the receptor and for binding of the non-peptide antagonists, respectively [33], [34]. Residue T270^7.43^A occupies a key position, is moderately conserved and is important for ligand interaction in GPCRs [35]. In rhodopsin, its cognate ligand (11-*cis*-retinal) is covalently attached to K296^7.43^, while, in AT1R, Y292^7.43^ is known to play a key role in the activation of AT1R signaling [36]–[39]. The I191^5.42^A mutant demonstrated a WT-like activation profile for both agonists, with slight bias for NPFF compared to AR-*agonist* in the calcium generation assay. The equivalent residue K199^5.42^ and M207^5.42^ in AT1R and rhodopsin, respectively, are reported to interact with their ligands and are shown to be involved in the rearrangement of hydrogen bond network between transmembrane helices 3 and 5 upon activation [32], [40]. Taken together, functional studies of these MAS mutants suggest that (1) peptide and non-peptide ligands share slightly different but overlapping binding sites and (2) the ligand binding regions in MAS are homologous to well-defined binding pockets in the transmembrane regions of rhodopsin and AT1R.

The concept that Ang(1–7) is an endogenous ligand is primarily based on the lack of response to Ang(1–7) and A779 treatment observed in mice and tissues that are deficient in MAS as compared to WT controls. Quality of radioligand binding and other pharmacological data reported to validate a direct interaction between Ang(1–7) and MAS is very poor. In our studies Ang(1–7) failed to activate G protein dependent pathways through MAS in the pharmacological concentration range. In agreement with our findings, there are reports that also show lack of G protein activation by MAS upon treatment with Ang(1–7) [17], [24], [25]. We experimentally ruled out the possibility that the *myc*-tag present in our MAS expression construct selectively compromises Ang(1–7) mediated signaling while not interfering with NPFF.

At supra-physiological concentrations (>100 µM) the response stimulated by Ang(1–7) was rapid calcium release without any IP1 production. Among various Ang(1–7) analogs, and related angiotensin fragments that we tested, none of the ligands strongly activated MAS. However, Sar^1^-Ang(1–7)-NH2, AngIII and AngIV-amide elicited MAS response with an EC_50_>10 µM. The faster kinetics of calcium flux and ability of MAS to be re-stimulated by Ang(1–7) analogs and related angiotensin fragments suggests an NPFF-like atypical signaling. Based on these observations we speculate that peptide ligands mediate atypical MAS signaling which is different from that of the non-peptides. Physiological significance of activation of MAS signaling at >100 µM concentration of Ang(1–7) analogs and related angiotensin fragments is unclear. However, it is important to recognize that high local concentration may exist in the intercellular space in vivo for autocrine/paracrine ligands.

Comparing the peptide sequences of AngIV-NH2, AngIII and Sar^1^-Ang(1–7)-NH2 that are positive hits in our screen with that of Ang(1–7) (Table 4), it appears that the (1) lack of aspartic residue (D) at the amino terminus, (2) presence of a hydrophobic residue (F) at the c-terminus and (3) amidation of carboxyl terminus are structural features favorable for activating MAS. Based on these observations it is conceivable that *in vivo* Ang(1–7) undergoes chemical modifications that promotes its binding to MAS and cause its activation.

It will be important to understand the molecular basis for the discrepancy in reports of MAS activation by Ang(1–7) in studies carried out in animals or isolated tissues and in transfected cell lines. Suggested possibilities include (1) modifications are required for Ang(1–7) to function through MAS [21]–[23] and (2) absence of allosteric proteins including other GPCRs that might sensitize the function of MAS to Ang(1–7) by assembling a tissue-specific ‘MAS-signalosome’. MAS and MRGs are reported to functionally couple to other GPCRs and ion channels providing reasonable evidence for the existence of ‘MAS-signalosomes’ [43], [52], [55]–[64] possibly in a cell-type dependent manner. Our future work is directed to examine these concepts for MAS and its ligands.

**Figure 7:**
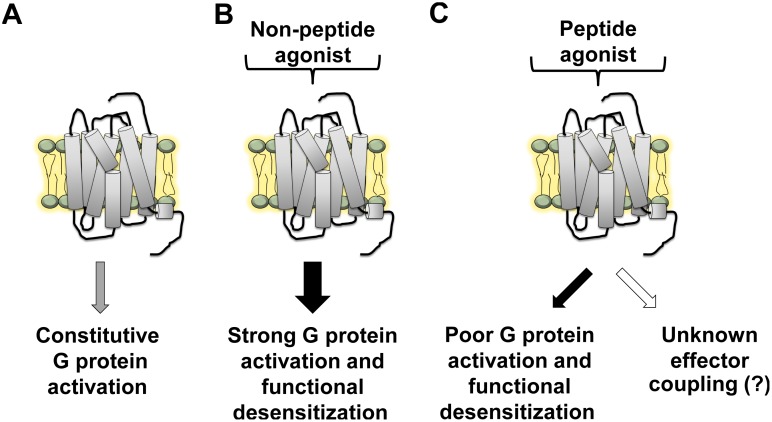
Schematic model for distinct signaling mechanisms of MAS. (A) Major G protein signaling pathways are constitutively activated by MAS. (B) Non-peptide agonist elevates MAS-mediated G protein activation beyond the constitutive activity and potentially promotes subsequent functional desensitization of MAS. (C) Physiological peptide agonists poorly activate MAS-mediated G protein activation beyond the constitutive activity and weakly promote functional desensitization of MAS. Strong calcium potentiation with very weak increase in intracellular IP1 levels along with unique calcium kinetics suggests that MAS possibly engages an unknown effector protein upon stimulation with peptide ligands.

Based on our data, we propose that a common profile of MAS signaling for peptide ligands may be weak coupling to classical G proteins in cells and poor desensitization. Constitutive activity of MAS and its activation by non-peptide ligands (AR-*agonist*) is typical of GPCR signaling (Figure 7A and 7B). Activation of MAS by the peptide ligand NPFF weakly activates G protein dependent and an unknown (possibly G protein independent) signaling pathway resulting in poor desensitization of the receptor (Figure 7C). Although, weak activators, there are similarities in the signaling and desensitization of MAS by Ang(1–7) analogs and angiotensin fragments compared to that of NPFF. Future efforts are focused on understanding the molecular basis of the complex signaling observed upon activation of MAS by physiological peptide ligands.

## Supporting Information

Figure S1 **Whole cell ELISA of WT and mutant MAS expressing stable cell lines.** Cell surface expression of MAS mutants relative to WT as quantitated by whole cell ELISA. The anti-c-*myc* (9E10) antibody (Santa Cruz Biotechnology, Inc. Santa Cruz, CA) was used for the ELISA. WT un-induced (UI) cells were included as a negative control. Data is presented as an average (mean±SEM) of two independent experiments (N = 2). In each experiment measurements are made at least in duplicate.
(TIF)

Figure S2 **Constitutive activity of MAS in G protein-mediated signaling.** Constitutive activity in induced cells expressing MAS compared to un-induced cells as measured by (A) calcium (B) IP1 (C) Luciferase and (D) cAMP assays. Data is presented as an average (mean±SEM) of multiple independent experiments. The number of independent experiments is: N>3 in panels A, B and C; N = 2 in panel D. Significance levels of *t*-test are given as: *p<0.05; **p<0.005; ***p<0.0001.
(TIF)

Figure S3 **Calcium and IP1 assay responses in WT MAS stable cell line in un-induced conditions (negative controls).** Dose dependent changes in (A) calcium flux (B) IP1 levels and (C) luciferase activity in cells upon stimulation with AR-*agonist*, AR-*inverse agonist* (AR-*inverse*), NPFF and Ang(1–7) in un-induced WT stable cell line. (D) Sar^1^-Ang(1–7)-NH2, AngIII and AngIV-NH2 dose-response curves in calcium assays for un-induced MAS stable cell. Representative curves from a single experiment wherein measurements are made in triplicate are shown as mean±SEM. The number of independent experiments is: N> = 2 in panels A and B; N = 1 in panels C and D.
(TIF)

Figure S4 **Dose-response for NPFF analogs in calcium assay.** Dose-response curves for NPFF and NPFF analogs - [Y^1^]-NPFF, [^D^Y^1^] [^NMe^F^3^]-NPFF and NPFF-C (see table 2 for amino acid sequence details of NPFF-analogs). Data is presented as mean±SEM from triplicate determinations from a representative experiment of at least three independent experiments (N = 3).
(TIF)

Figure S5 **Real-time quantitative PCR (qPCR) verification of** ***MAS*** **,** ***NPFFR1*** **and** ***NPFFR2*** **gene expression.** The log-fold increase in gene expression (2^−ΔΔCt^) of genes in un-induced and induced WT-MAS stable cells are shown compared to parental HEK293 cells from which the MAS cell lines were established. The expression of *RRN18S* is used as an endogenous internal control. Data is presented as an average (mean±SEM) of two independent experiments (N = 2). In each experiment measurements are made in triplicate. Significance levels of *t*-test are given as: *p<0.05; n.s., not significant.
(TIF)

Figure S6 **Constitutive activity in wild-type (WT) and mutant MAS stable cell lines.** The basal IP1 levels (in the absence of any ligand treatment) for WT and mutant stable cell lines are corrected for cell surface expression relative to the WT and are shown as bar graphs. The horizontal dashed lines indicate the IP1 levels in WT un-induced (UI) stable cells. The shaded region indicates the range of basal/constitutive activity observed in the WT and mutant MAS cell lines. Data from multiple independent experiments (N> = 3) wherein measurements are made in triplicate are presented as mean±SEM. Significance levels of *t*-test are given as: *p<0.05; **p<0.005.
(TIF)

Figure S7 **IP1 assays in un-induced mutant MAS stable cell lines.** The dose response curves for (A) AR-*inverse agonist* (AR-*inverse*), (B) AR-*agonist* and (C) NPFF are measured as function of IP1 levels in the cells. Representative curves from a single experiment wherein measurements are made in triplicates are shown as mean±SEM. The number of independent experiments is: N> = 2 in panels A and B; N = 1 in panel C.
(TIF)

Figure S8 **Calcium assay responses in untagged WT MAS stable cell line.** Dose dependent changes in calcium flux in cells upon stimulation with AR-*agonist*, AR-*inverse agonist* (AR-*inverse*), NPFF and Ang(1–7). The EC_50_ values for AR-*agonist* and NPFF are 1.2±0.1 µM and 0.4±0.1 µM, respectively, while the IC_50_ value for AR-*inverse* is 0.5±0.4 µM. Ang(1–7) treatment shows no response. Data is presented as mean±SEM from triplicate determinations from a representative experiment of at least three independent experiments (N = 3).
(TIF)

Figure S9 **Screening of Ang(1–7) analogs and angiotensin metabolites (in** **table 4** **) in a modified calcium assay re-stimulation protocol.** MAS expressing stable cells were first treated with (A) No ligand (control) and with test ligands (B) Ang(1–7), (C) A779, (D) Sar^1^-Ang(1–7)-NH2, (E) AngIII, (F) AngIV-NH2, (G) AngIV and (H) Ang(3–7) followed by re-stimulation with NPFF. The screen identified Sar^1^-Ang(1–7)-NH2, AngIII and AngIV-NH2 as weak agonists. Data is presented as mean±SEM from duplicate determinations from one independent experiment (N = 1).
(TIF)

Table S1 **List of ligands reported to activate MAS.**
(DOC)

Table S2 **Basal IP1 levels (not corrected for cell surface expression) in un-induced and induced wild-type** **(WT) and mutant MAS stable cell lines.**
(DOC)

Methods S1 **The protocols used for enzyme-linked immunosorbent assay (ELISA), RNA isolation, cDNA preparation, real-time quantitative PCR (qPCR) and the measurement of intracellular cAMP are described.**
(DOC)
