# Supplementary figures and images for: Atypical Signaling and Functional Desensitization Response of MAS Receptor to Peptide Ligands
Source: PLoS One. 2014 Jul 28;9(7):e103520. doi: 10.1371/journal.pone.0103520 (PMC4113456; doi:10.1371/journal.pone.0103520)

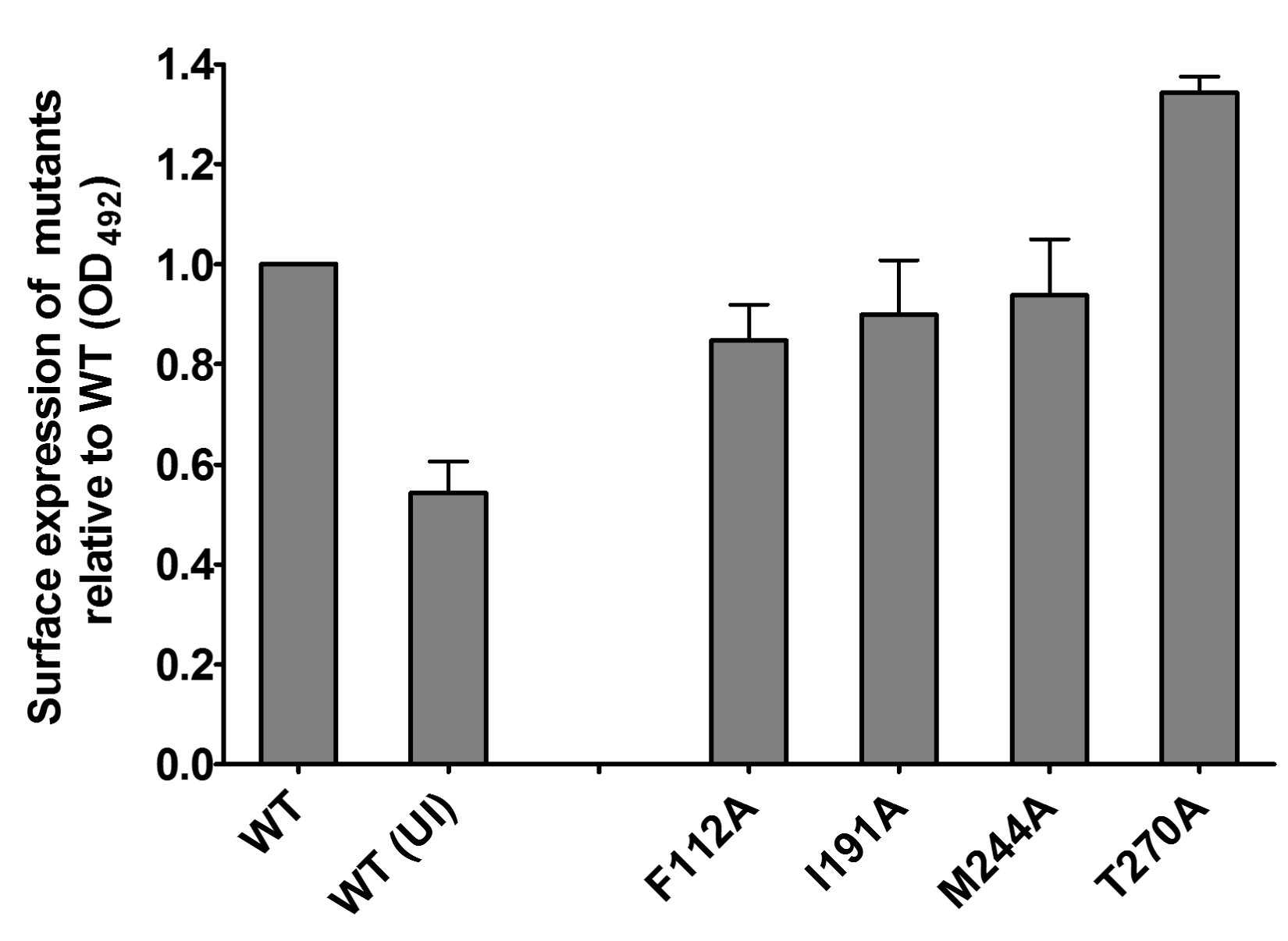

Supplement: Figure S1 — Whole cell ELISA of WT and mutant MAS expressing stable cell lines. Cell surface expression of MAS mutants relative to WT as quantitated by whole cell ELISA. The anti-c-myc (9E10) antibody (Santa Cruz Biotechnology, Inc. Santa Cruz, CA) was used for the ELISA. WT un-induced (UI) cells were included as a negative control. Data is presented as an average (mean±SEM) of two independent experiments (N = 2). In each experiment measurements are made at least in duplicate. (TIF) [file pone.0103520.s001.tif]

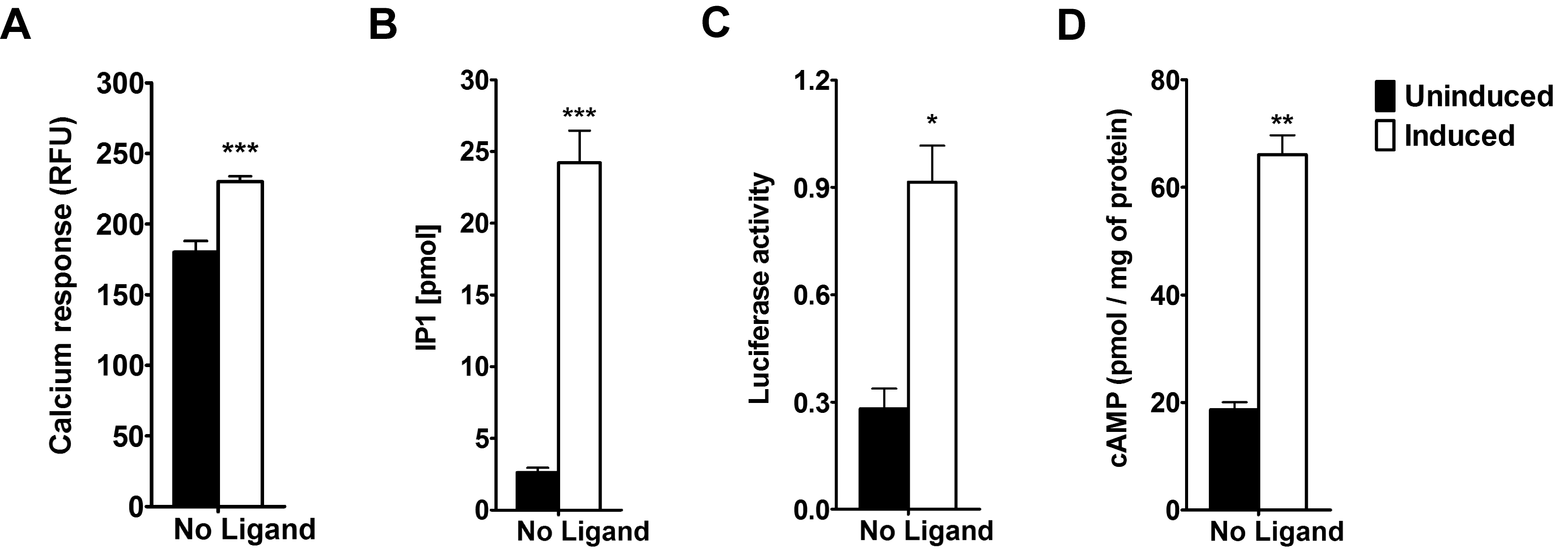

Supplement: Figure S2 — Constitutive activity of MAS in G protein-mediated signaling. Constitutive activity in induced cells expressing MAS compared to un-induced cells as measured by (A) calcium (B) IP1 (C) Luciferase and (D) cAMP assays. Data is presented as an average (mean±SEM) of multiple independent experiments. The number of independent experiments is: N>3 in panels A, B and C; N = 2 in panel D. Significance levels of t-test are given as: *p<0.05; **p<0.005; ***p<0.0001. (TIF) [file pone.0103520.s002.tif]

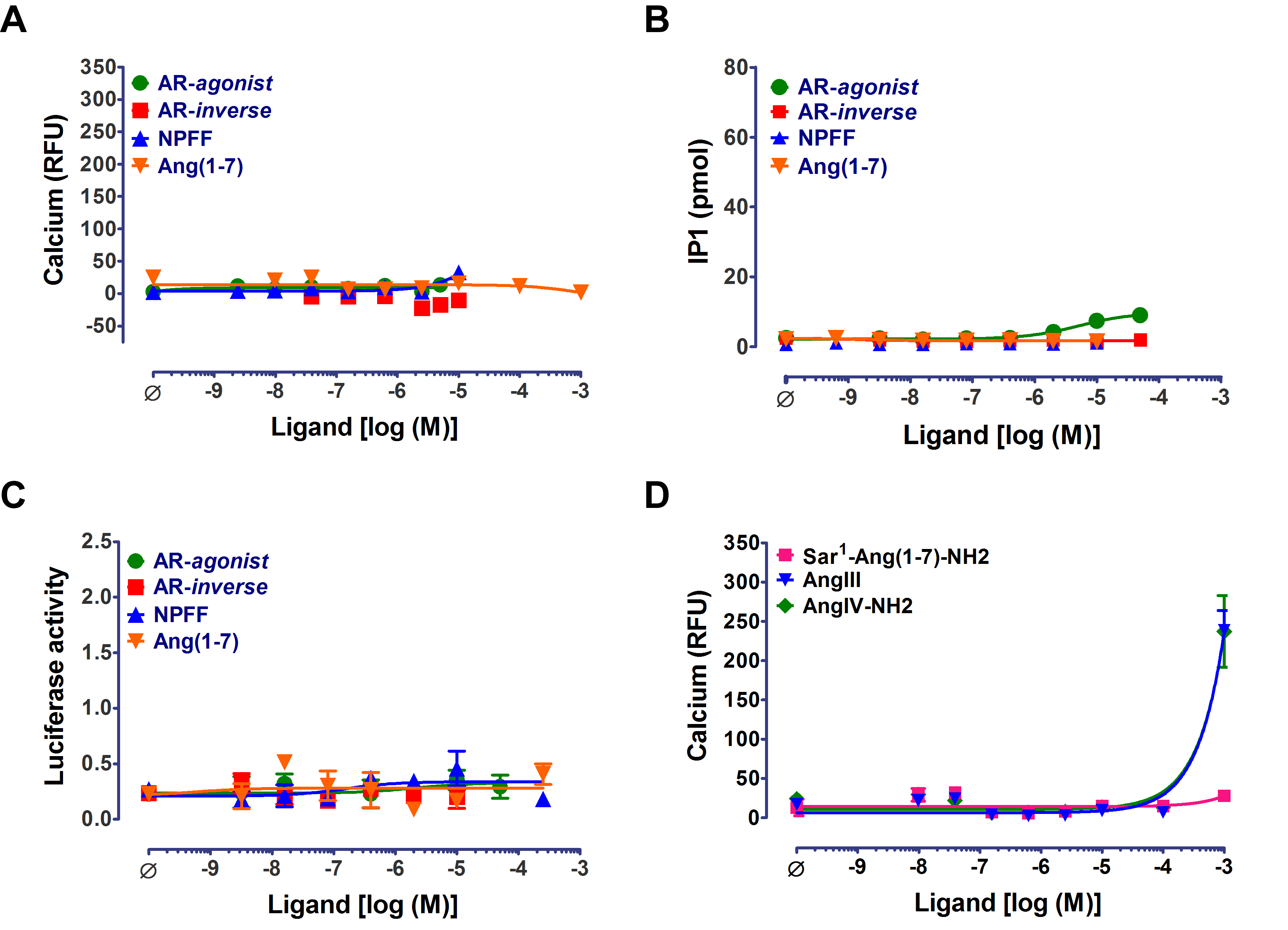

Supplement: Figure S3 — Calcium and IP1 assay responses in WT MAS stable cell line in un-induced conditions (negative controls). Dose dependent changes in (A) calcium flux (B) IP1 levels and (C) luciferase activity in cells upon stimulation with AR-agonist, AR-inverse agonist (AR-inverse), NPFF and Ang(1–7) in un-induced WT stable cell line. (D) Sar1-Ang(1–7)-NH2, AngIII and AngIV-NH2 dose-response curves in calcium assays for un-induced MAS stable cell. Representative curves from a single experiment wherein measurements are made in triplicate are shown as mean±SEM. The number of independent experiments is: N> = 2 in panels A and B; N = 1 in panels C and D. (TIF) [file pone.0103520.s003.tif]

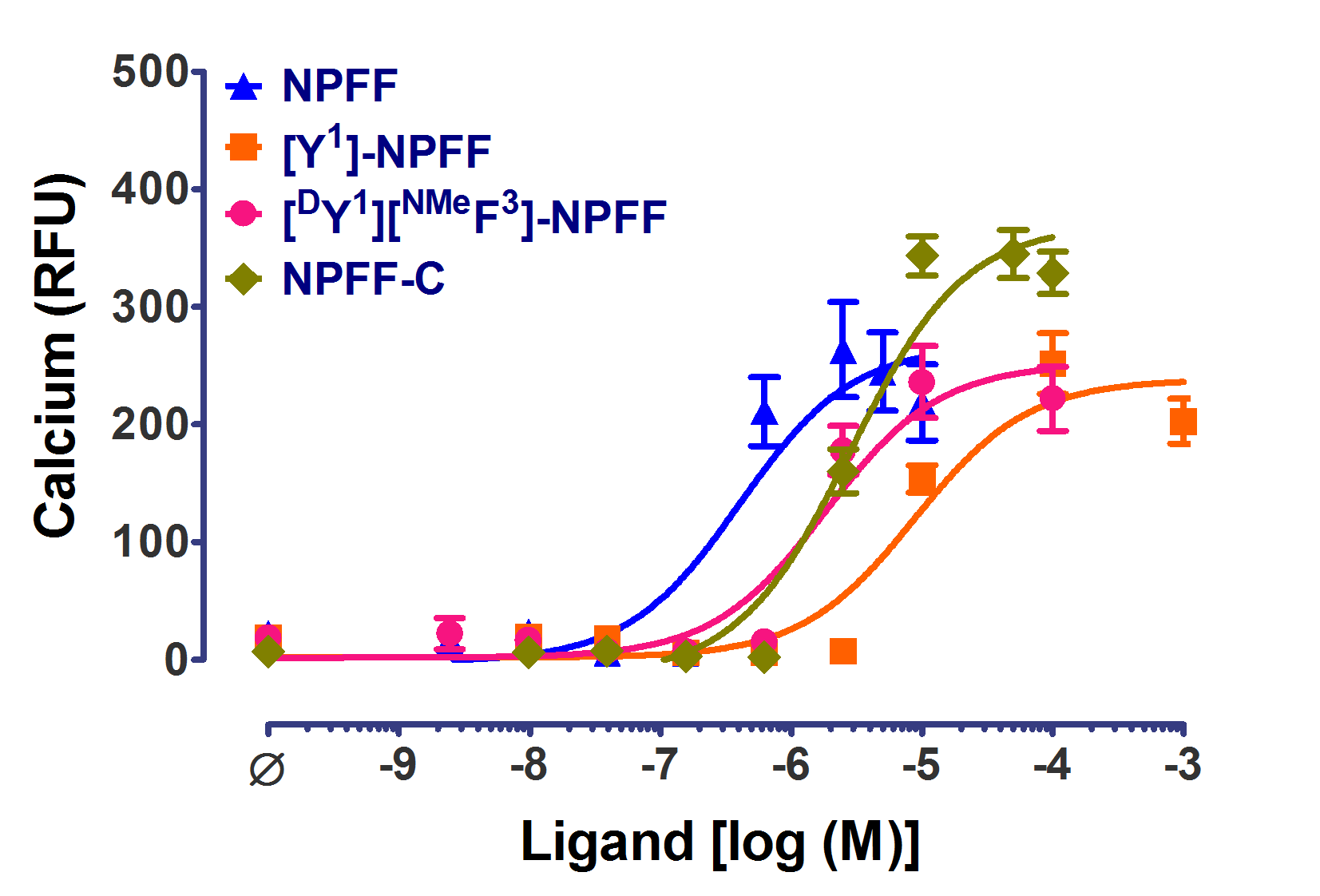

Supplement: Figure S4 — Dose-response for NPFF analogs in calcium assay. Dose-response curves for NPFF and NPFF analogs - [Y1]-NPFF, [DY1] [NMeF3]-NPFF and NPFF-C (see table 2 for amino acid sequence details of NPFF-analogs). Data is presented as mean±SEM from triplicate determinations from a representative experiment of at least three independent experiments (N = 3). (TIF) [file pone.0103520.s004.tif]

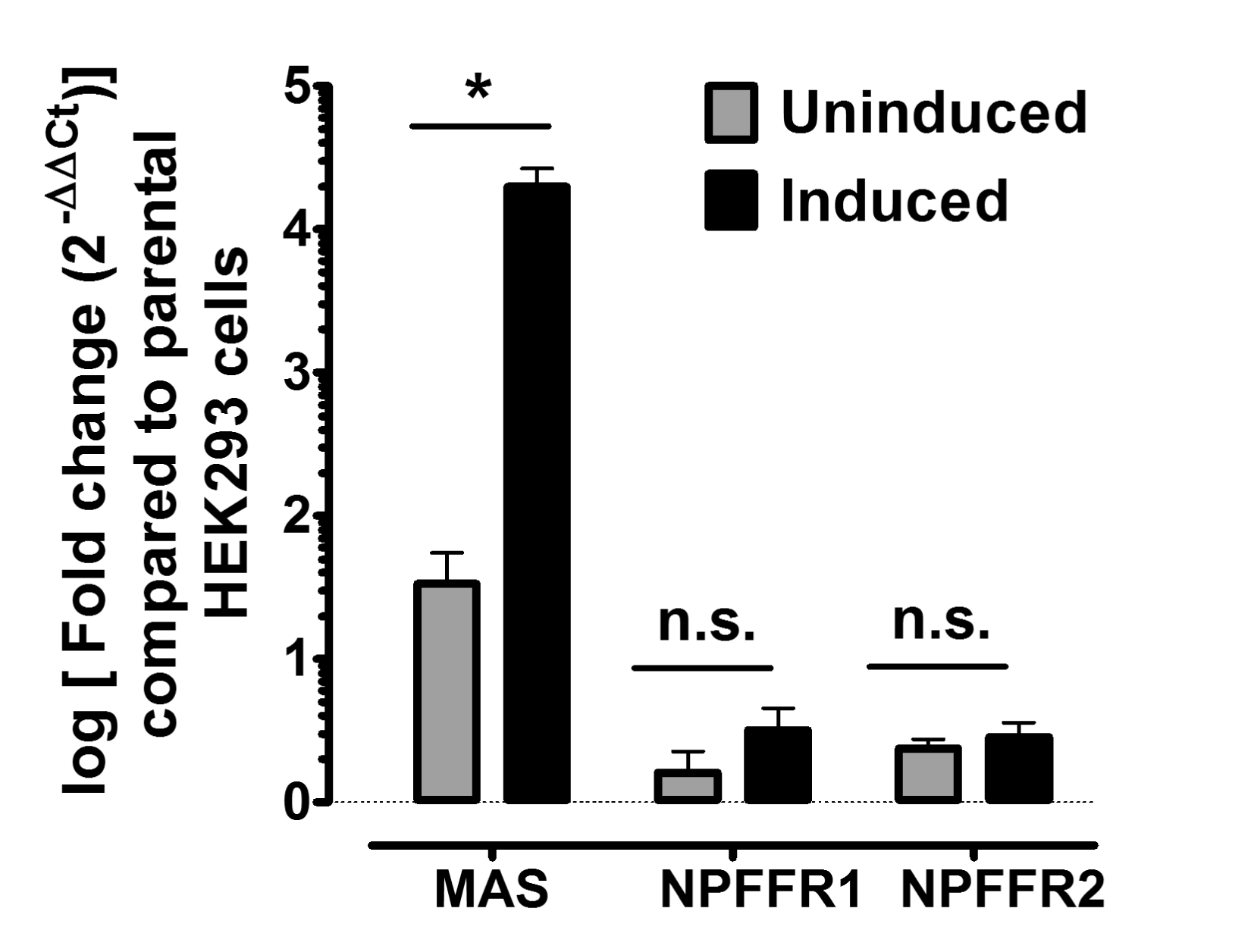

Supplement: Figure S5 — Real-time quantitative PCR (qPCR) verification of MAS , NPFFR1 and NPFFR2 gene expression. The log-fold increase in gene expression (2−ΔΔCt) of genes in un-induced and induced WT-MAS stable cells are shown compared to parental HEK293 cells from which the MAS cell lines were established. The expression of RRN18S is used as an endogenous internal control. Data is presented as an average (mean±SEM) of two independent experiments (N = 2). In each experiment measurements are made in triplicate. Significance levels of t-test are given as: *p<0.05; n.s., not significant. (TIF) [file pone.0103520.s005.tif]

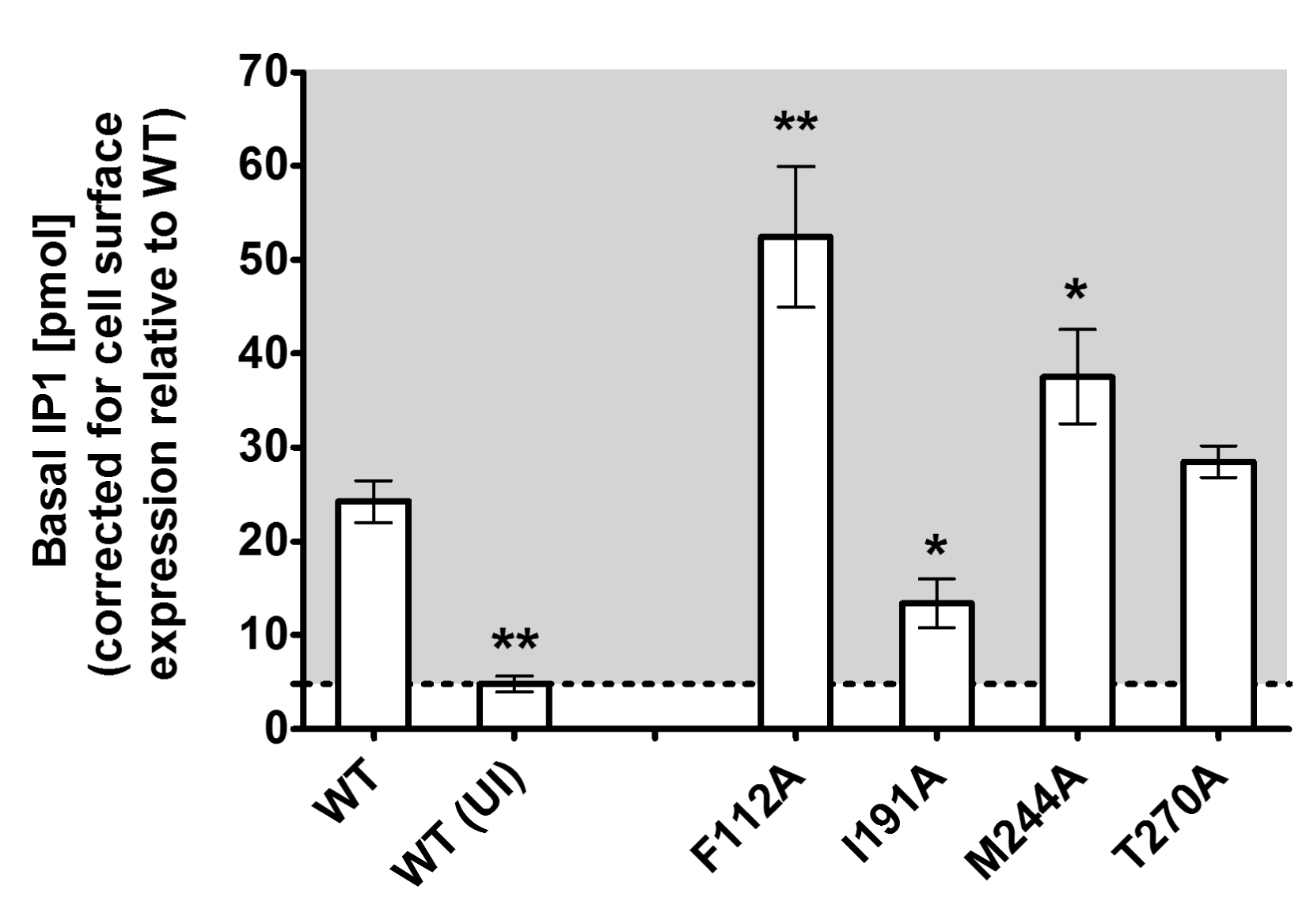

Supplement: Figure S6 — Constitutive activity in wild-type (WT) and mutant MAS stable cell lines. The basal IP1 levels (in the absence of any ligand treatment) for WT and mutant stable cell lines are corrected for cell surface expression relative to the WT and are shown as bar graphs. The horizontal dashed lines indicate the IP1 levels in WT un-induced (UI) stable cells. The shaded region indicates the range of basal/constitutive activity observed in the WT and mutant MAS cell lines. Data from multiple independent experiments (N> = 3) wherein measurements are made in triplicate are presented as mean±SEM. Significance levels of t-test are given as: *p<0.05; **p<0.005. (TIF) [file pone.0103520.s006.tif]

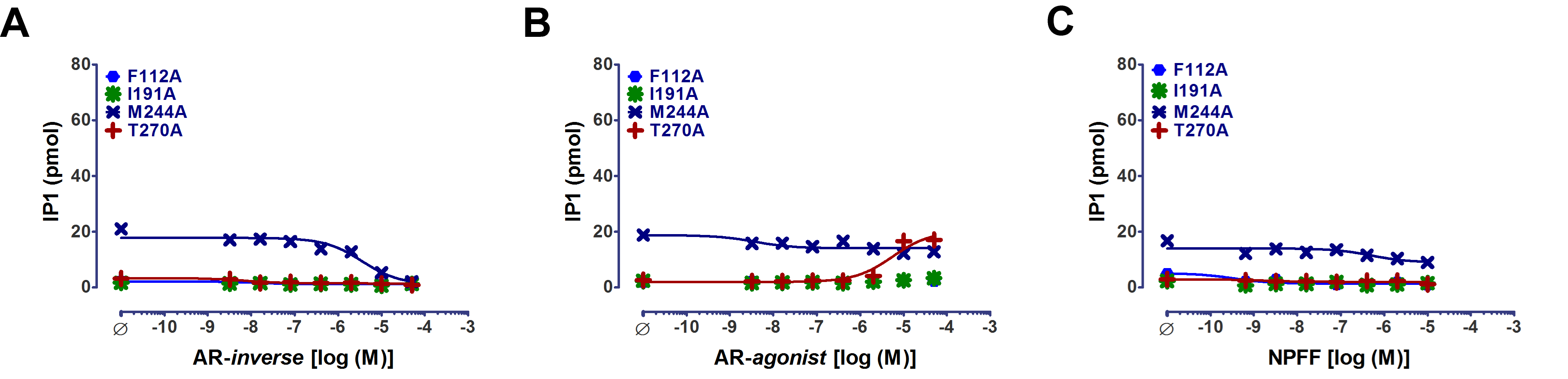

Supplement: Figure S7 — IP1 assays in un-induced mutant MAS stable cell lines. The dose response curves for (A) AR-inverse agonist (AR-inverse), (B) AR-agonist and (C) NPFF are measured as function of IP1 levels in the cells. Representative curves from a single experiment wherein measurements are made in triplicates are shown as mean±SEM. The number of independent experiments is: N> = 2 in panels A and B; N = 1 in panel C. (TIF) [file pone.0103520.s007.tif]

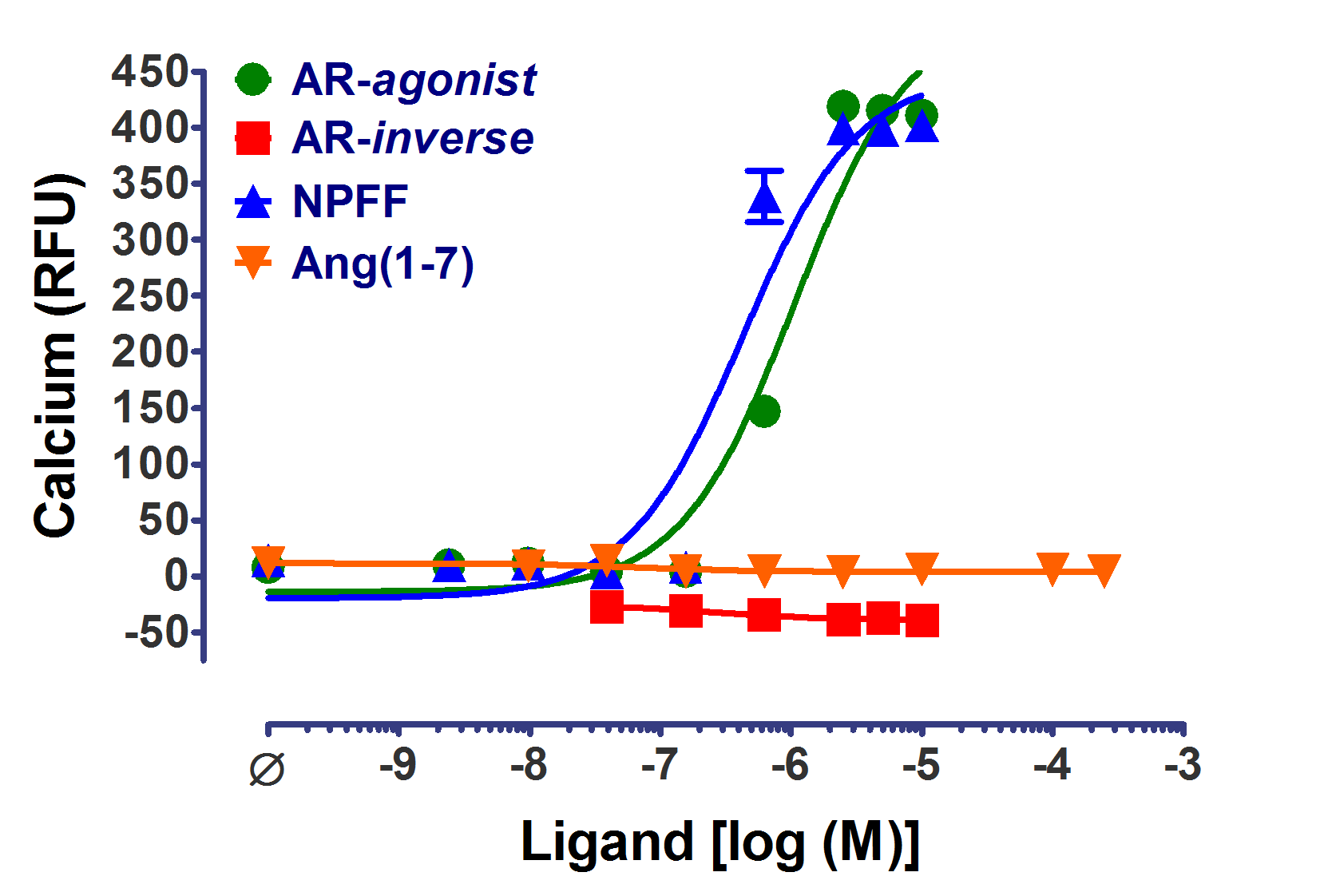

Supplement: Figure S8 — Calcium assay responses in untagged WT MAS stable cell line. Dose dependent changes in calcium flux in cells upon stimulation with AR-agonist, AR-inverse agonist (AR-inverse), NPFF and Ang(1–7). The EC50 values for AR-agonist and NPFF are 1.2±0.1 µM and 0.4±0.1 µM, respectively, while the IC50 value for AR-inverse is 0.5±0.4 µM. Ang(1–7) treatment shows no response. Data is presented as mean±SEM from triplicate determinations from a representative experiment of at least three independent experiments (N = 3). (TIF) [file pone.0103520.s008.tif]

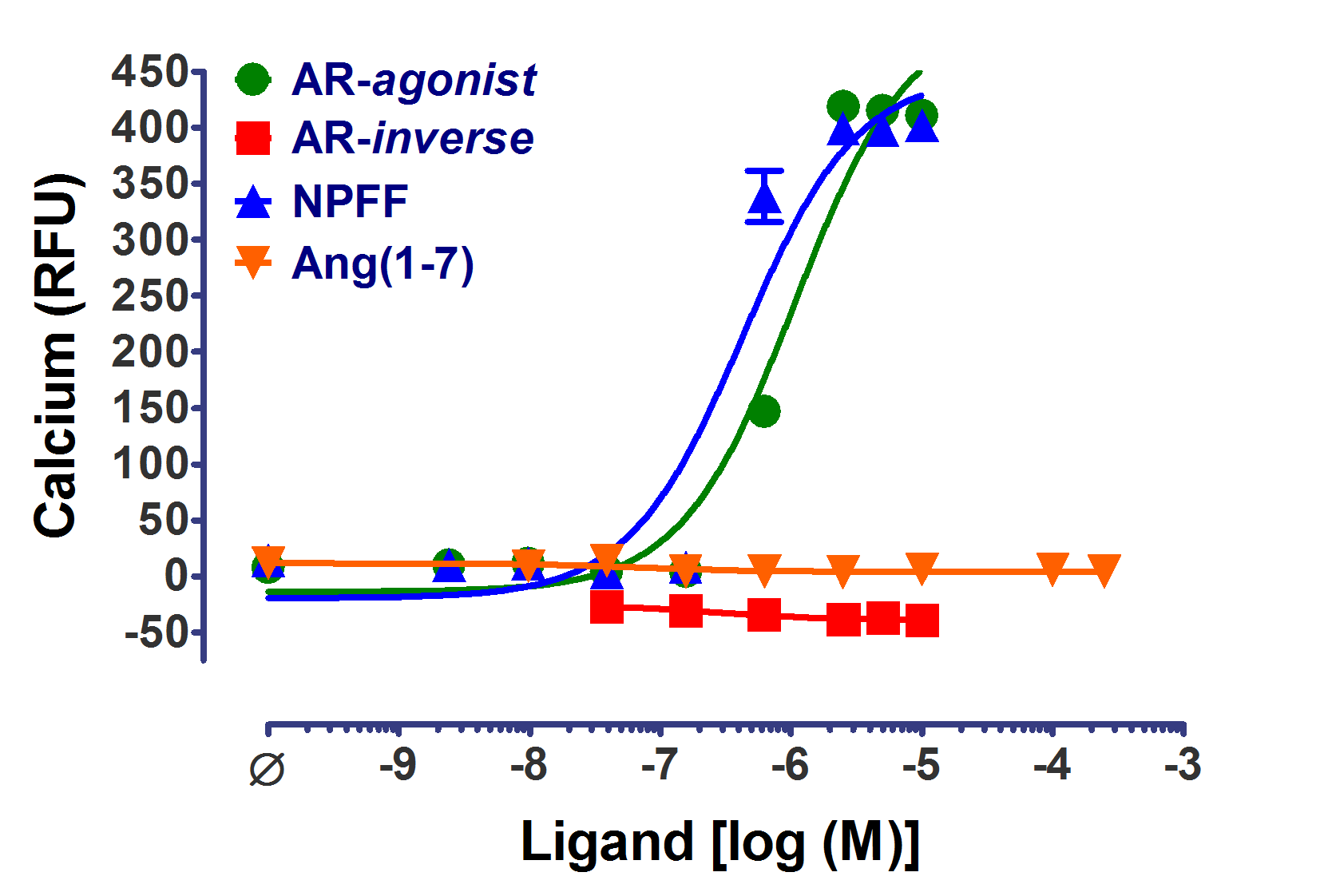

Supplement: Figure S9 — Screening of Ang(1–7) analogs and angiotensin metabolites (in table 4 ) in a modified calcium assay re-stimulation protocol. MAS expressing stable cells were first treated with (A) No ligand (control) and with test ligands (B) Ang(1–7), (C) A779, (D) Sar1-Ang(1–7)-NH2, (E) AngIII, (F) AngIV-NH2, (G) AngIV and (H) Ang(3–7) followed by re-stimulation with NPFF. The screen identified Sar1-Ang(1–7)-NH2, AngIII and AngIV-NH2 as weak agonists. Data is presented as mean±SEM from duplicate determinations from one independent experiment (N = 1). (TIF) [file pone.0103520.s009.tif]
