# Supplementary material for: Atypical Signaling and Functional Desensitization Response of MAS Receptor to Peptide Ligands
Source: PLoS One. 2014 Jul 28;9(7):e103520. doi: 10.1371/journal.pone.0103520 (PMC4113456; doi:10.1371/journal.pone.0103520)
Supplement: Table S1 — List of ligands reported to activate MAS. (DOC) [file pone.0103520.s010.doc]

**Table S1. List of ligands reported to activate MAS.**

| **Ligand name** | **Sequence** | **Type** | **Remarks** | **MAS** | **Ref** |
| --- | --- | --- | --- | --- | --- |
| **Physiological ligands** | | | | | |
| **Neuropeptide FF** | FLFQPQRF-amide | Agonist | EC_50_ = 400nM (Calcium assay) | Mouse | [1] |
| **Angiotensin (1-7)** | DRVYIHP | Endogenous agonist | Arachidonic acid release assay | Mouse | [2] |
|  |  |  | Vasodilator effect in aortic rings | Mouse | [2] |
| **Angiotensin III** | RVYIHPF | Agonist | Arachidonic acid release assay | Human | [3] |
| **Angiotensin IV** | VYIHPF | Agonist | Arachidonic acid release assay | Human | [3] |
| **Angioprotectin** | PEVYIHPF | Agonist | Vasodilator effect in aortic rings | Human | [4] |
| **Derivatives of Angiotensin (1-7)** | | | | | |
| **A779** | [^D^Ala^7^]-Ang(1-7) | Antagonist | Inhibits Ang(1-7) response | Mouse | [2,5] |
| **Selective antagonist** | [^D^Pro^7^]-Ang(1-7) | Antagonist | Inhibits Ang(1-7) response | Mouse | [6] |
| **Cyclized Ang(1-7)** | Modified Ang(1-7) | Agonist | Resistant to proteolysis | Rat | [7] |
| **AVE 0991** | Non-peptide mimic | Agonist | Mimics Ang(1-7) effects | Bovine | [8] |
| **Other ligands** | | | | | |
| **AR234960** | Non-peptide | Agonist | EC_50_ = 0.35µM (IP1 assay) | Human | [9] |
|  |  |  | EC_50_ = 0.72µM (cAMP assay) | Human | [9] |
|  |  |  | EC_50_ = 0.17µM (IP1 assay) | Rat | [9] |
|  |  |  | EC_50_= 1.71µM (cAMP assay) | Rat | [9] |
| **AR244555** | Non-peptide | Inverse Agonist | EC_50_ = 0.186µM (IP1assay) | Human | [9] |
|  |  |  | EC_50_ = 0.348µM (IP1 assay) | Rat | [9] |
| **AR305352** | Non-peptide | Inverse Agonist | EC_50_ = 0.166µM (IP1 assay) | Human | [9] |
|  |  |  | EC_50_ = 0.320µM (IP1 assay) | Rat | [9] |
| **P33 / CGEN-857** | SMCHRWSRAVLFPAAHRP | Agonist | EC_50_ = 1µM (Calcium assay) | Human | [10,11] |
| **P61 / CGEN-856** | FLGYCIYLNRKRRGDPAFKRRLRD | Agonist | EC_50_= 0.57µM (Calcium assay) | Human | [10,11] |
| **MBP7** | KAQLRRLS | Agonist | EC_50_ = 36.41µM (Calcium assay) | Rat | [12] |

**References**

1. Dong X, Han S, Zylka MJ, Simon MI, Anderson DJ (2001) A diverse family of GPCRs expressed in specific subsets of nociceptive sensory neurons. Cell 106: 619-632.

2. Santos RA, Simoes e Silva AC, Maric C, Silva DM, Machado RP, et al. (2003) Angiotensin-(1-7) is an endogenous ligand for the G protein-coupled receptor Mas. Proc Natl Acad Sci U S A 100: 8258-8263.

3. Gembardt F, Grajewski S, Vahl M, Schultheiss HP, Walther T (2008) Angiotensin metabolites can stimulate receptors of the Mas-related genes family. Mol Cell Biochem 319: 115-123.

4. Jankowski V, Tolle M, Santos RA, Gunthner T, Krause E, et al. (2011) Angioprotectin: an angiotensin II-like peptide causing vasodilatory effects. FASEB J 25: 2987-2995.

5. Santos RA, Campagnole-Santos MJ, Baracho NC, Fontes MA, Silva LC, et al. (1994) Characterization of a new angiotensin antagonist selective for angiotensin-(1-7): evidence that the actions of angiotensin-(1-7) are mediated by specific angiotensin receptors. Brain Res Bull 35: 293-298.

6. Santos RA, Haibara AS, Campagnole-Santos MJ, Simoes e Silva AC, Paula RD, et al. (2003) Characterization of a new selective antagonist for angiotensin-(1-7), D-pro7-angiotensin-(1-7). Hypertension 41: 737-743.

7. Kluskens LD, Nelemans SA, Rink R, de Vries L, Meter-Arkema A, et al. (2009) Angiotensin-(1-7) with thioether bridge: an angiotensin-converting enzyme-resistant, potent angiotensin-(1-7) analog. J Pharmacol Exp Ther 328: 849-854.

8. Wiemer G, Dobrucki LW, Louka FR, Malinski T, Heitsch H (2002) AVE 0991, a nonpeptide mimic of the effects of angiotensin-(1-7) on the endothelium. Hypertension 40: 847-852.

9. Zhang T, Li Z, Dang H, Chen R, Liaw C, et al. (2012) Inhibition of Mas G-protein signaling improves coronary blood flow, reduces myocardial infarct size, and provides long-term cardioprotection. Am J Physiol Heart Circ Physiol 302: H299-311.

10. Shemesh R, Toporik A, Levine Z, Hecht I, Rotman G, et al. (2008) Discovery and validation of novel peptide agonists for G-protein-coupled receptors. J Biol Chem 283: 34643-34649.

11. Savergnini SQ, Beiman M, Lautner RQ, de Paula-Carvalho V, Allahdadi K, et al. (2010) Vascular relaxation, antihypertensive effect, and cardioprotection of a novel peptide agonist of the MAS receptor. Hypertension 56: 112-120.

12. Bikkavilli RK, Tsang SY, Tang WM, Sun JX, Ngai SM, et al. (2006) Identification and characterization of surrogate peptide ligand for orphan G protein-coupled receptor mas using phage-displayed peptide library. Biochem Pharmacol 71: 319-337.
