# Supplementary material for: Atypical Signaling and Functional Desensitization Response of MAS Receptor to Peptide Ligands
Source: PLoS One. 2014 Jul 28;9(7):e103520. doi: 10.1371/journal.pone.0103520 (PMC4113456; doi:10.1371/journal.pone.0103520)
Supplement: Table S2 — Basal IP1 levels (not corrected for cell surface expression) in un-induced and induced wild-type (WT) and mutant MAS stable cell lines. (DOC) [file pone.0103520.s011.doc]

**Table S2. Basal IP1 levels (not corrected for cell surface expression) in un-induced and induced wild-type (WT) and mutant MAS stable cell lines.**

|  | **Basal IP1 levels [pmol]** | |
| --- | --- | --- |
| **MAS constructs** | **Un-induced** | **Induced** |
| **WT** | 2.6±0.3 | 24.2±2.2******* |
| **Ligand binding domain mutants** | | |
| **F112A** | 2.8±0.7 | 44.5±5.2****** |
| **I191A** | 1.7±0.3 | 12.0±1.8****** |
| **M244A** | 16.6±1.7 | 35.2±2.0******* |
| **T270A** | 2.9±0.6 | 38.3±2.1******* |

Values are mean±SEM from at least three independent experiments; Statistical significance (t-test) - *p<0.05, **p<0.005, ***p<0.0001.
