# Supplementary material for: Atypical Signaling and Functional Desensitization Response of MAS Receptor to Peptide Ligands
Source: PLoS One. 2014 Jul 28;9(7):e103520. doi: 10.1371/journal.pone.0103520 (PMC4113456; doi:10.1371/journal.pone.0103520)
Supplement: Methods S1 — The protocols used for enzyme-linked immunosorbent assay (ELISA), RNA isolation, cDNA preparation, real-time quantitative PCR (qPCR) and the measurement of intracellular cAMP are described. (DOC) [file pone.0103520.s012.doc]

**Methods S1**

**Enzyme-linked immunosorbent assay (ELISA) to quantify receptor expression on cell surface.** The protocol for cell surface ELISA is adapted from previous reports on AT1R and β_2_-adrenergic receptor [1,2]. In brief, 1.25 million cells per well in 1ml complete media were seeded in a 12 well plate that was pre-coated with poly-L-lysine. The plates were left in a cell culture incubator overnight. On the following day, cells were treated with induction media (complete media for negative controls) and the ELISA was performed 26-28h post-induction. Following induction, cells were placed on ice (to prevent endocytosis of the receptor during processing of the samples) and washed twice with 1ml HBSS/1% bovine serum albumin (BSA). The cells were then incubated on ice for 1h with anti-c-*myc* (9E10) antibody (Santa Cruz Biotechnology, Inc. Santa Cruz, CA) diluted in HBSS/1%BSA to 1µg/ml. This was followed by two more washes with HBSS/1%BSA and the cells were fixed with 4% paraformaldehyde in HBSS for 15min on ice. The cells were washed 2 times with HBSS/0.5%BSA and incubated with anti-mouse HRP conjugated secondary antibody (GE Healthcare, UK) at a dilution of 1:1000 for 1h at room temperature. Cells were then washed twice with HBSS/0.5%BSA and finally incubated with 400µl of OPD substrate (o-Phenylenediamine dihydrochloride; Sigma-Aldrich, St. Louis, MO) for 10min. The reaction was stopped by transferring 200µl of reaction to a 96 well plate with 50µl of 3N HCl. The absorption readings from the 96 well plates were measured at 492nm using FlexStation 3 instrument (Molecular Devices, Sunnyvale, CA).

**RNA isolation, cDNA preparation and real-time quantitative PCR (qPCR) analysis.** The gene expression levels of 18S ribosomal RNA (*RRN18S*; used as an internal control), *MAS*, *neuropeptide FF* *receptor 1* (*NPFFR2*) and *receptor 2* (*NPFFR2*) were evaluated by qPCR. Total RNA from T-Rex™ HEK293 cells (parental cells from Life Technologies, Grand Island, NY used to establish inducible stable cells) and un-induced and induced WT-MAS stable cell lines was isolated from cell pellets prepared from 10cm plates using the miRNeasy Mini Kit from Qiagen (Cat. No. 217004) under RNase free conditions, according to manufacturer’s protocol. Preparation of cDNA was done from 1µg of total template RNA using the iScript™ Reverse transcription kit from Bio-Rad (Cat. No. 170-8840) following the manufacturer’s protocol. For the qPCR, 20ng of cDNA preparation was mixed with 2x iQ™ SYBR Green Supermix from Bio-Rad (Cat. No. 170-8880) along with specific primers from Qiagen for human *RRN18S* (Cat. No. QT00199367), *NPFFR1* (Cat. No. QT00041363), *NPFFR2* (Cat. No. QT00016877) and *MAS* (Cat. No. QT00204302). The qPCR reaction was run on MyiQ™2 Two-Color Real-Time PCR Detection System from BioRad and the threshold cycle values (C_t_) and melt-curve analysis were recorded on the instrument. The fold increase in gene expression (2^-ΔΔCt^) of respective genes in un-induced and induced WT-MAS stable cells were calculated by comparing C_t_ values from T-Rex™ HEK293 cells (parental cells) following normalization to RRN18S.

**Measurement of intracellular cAMP**. The intracellular cAMP levels were measured using CatchPoint™ cAMP Fluorescent assay kit from Molecular devices (Molecular Devices, Sunnyvale, CA). For the cAMP assay, the cells were plated at a density of 1.25 million cells/well in a six-well plate. The cells were maintained in a cell culture incubator and induced the following day with doxycycline. The cells were serum starved for 2h following the induction for 26-28h. At this point the serum free media was replaced with 1ml of 3-Isobutyl-1-methylxanthine (Sigma-Aldrich, St. Louis, MO) at 1mM concentration for 15min at 37°C. Following this, 250µl of ligands were added at 5x concentration to the wells and incubated for another 15min at 37°C. The plates were then immediately transferred to ice and the cells were lysed with 150µl of lysis buffer provided with kit. The cAMP levels in the lysed samples were measured according to the protocol provided by the supplier.

**References**

1. Bonde MM, Hansen JT, Sanni SJ, Haunso S, Gammeltoft S, et al. (2010) Biased signaling of the angiotensin II type 1 receptor can be mediated through distinct mechanisms. PLoS One 5: e14135.

2. Makita N, Kabasawa Y, Otani Y, Firman, Sato J, et al. (2013) Attenuated desensitization of beta-adrenergic receptor by water-soluble N-nitrosamines that induce S-nitrosylation without NO release. Circ Res 112: 327-334.
